# Pluripotency gene network dynamics: System views from parametric analysis

**DOI:** 10.1371/journal.pone.0194464

**Published:** 2018-03-29

**Authors:** Ilya R. Akberdin, Nadezda A. Omelyanchuk, Stanislav I. Fadeev, Natalya E. Leskova, Evgeniya A. Oschepkova, Fedor V. Kazantsev, Yury G. Matushkin, Dmitry A. Afonnikov, Nikolay A. Kolchanov

**Affiliations:** 1 Federal Research Center Institute of Cytology and Genetics SB RAS, Novosibirsk, Russia; 2 Novosibirsk State University, Novosibirsk, Russia; 3 San Diego State University, San Diego, CA, United States of America; 4 Sobolev Institute of Mathematics SB RAS, Novosibirsk, Russia; Macau University of Science and Technology, MACAO

## Abstract

Multiple experimental data demonstrated that the core gene network orchestrating self-renewal and differentiation of mouse embryonic stem cells involves activity of Oct4, Sox2 and Nanog genes by means of a number of positive feedback loops among them. However, recent studies indicated that the architecture of the core gene network should also incorporate negative Nanog autoregulation and might not include positive feedbacks from Nanog to Oct4 and Sox2. Thorough parametric analysis of the mathematical model based on this revisited core regulatory circuit identified that there are substantial changes in model dynamics occurred depending on the strength of Oct4 and Sox2 activation and molecular complexity of Nanog autorepression. The analysis showed the existence of four dynamical domains with different numbers of stable and unstable steady states. We hypothesize that these domains can constitute the checkpoints in a developmental progression from naïve to primed pluripotency and vice versa. During this transition, parametric conditions exist, which generate an oscillatory behavior of the system explaining heterogeneity in expression of pluripotent and differentiation factors in serum ESC cultures. Eventually, simulations showed that addition of positive feedbacks from Nanog to Oct4 and Sox2 leads mainly to increase of the parametric space for the naïve ESC state, in which pluripotency factors are strongly expressed while differentiation ones are repressed.

## Introduction

Pluripotency is a temporal state in embryogenesis artificially maintained in embryonic stem cells (ESCs) by specific components in the medium providing ESC self-renewal and inhibition of signaling pathways driving differentiation (reviewed in [1, 2, 3]). In mouse pluripotent cells there are three types of media used for this, 2i/LIF, serum/LIF and FGF2/Activin providing for naïve, formative and primed pluripotent states, respectively. These states differ in the expression of pluripotency and differentiation genes with naïve and primed states biased correspondingly to pluripotency and differentiation. Somatic cells may be also driven to pluripotency on specific media, and obtained induced pluripotent stem cells (iPSCs) are very similar to ESCs in their molecular and functional traits [4], (reviewed in [5, 6]).

Along with whole-genome technologies, high-resolution imaging and other experimental approaches, mathematical modeling becomes one of the tools for understanding of molecular mechanisms behind cell pluripotency and differentiation (reviewed in [7, 8]). For simulations, a molecular genetic process represents as a regulatory gene network handling of input signals. Multiple experimental studies demonstrate that Oct4, Sox2 and Nanog (OSN) are the core factors in pluripotency gene network involved in induction, maintenance and loss of pluripotency (reviewed in [2, 9]).

Chickarmane and coauthors developed the first model of this core circuit and follow-up series of extended models to investigate a landscape of possible embryonic stem cell (ESC) states [10, 11, 12]. Already analysis of the first model, where Oct4, Sox2 and Nanog (OSN) regulated by environmental signals activate each other, indicated that the positive-feedback loops in the OSN circuit give rise to bistability, which corresponds to existence of two stable cell states (self-renewal/pluripotency and differentiation) toggle-switched by external signals [10]. Mutual regulation within OSN core were withdrawn from whole genome experiments on identification of OSN targets in both human ESCs (hESCs) and mESCs [13, 14]. Positive regulation of Nanog, Sox2 and Oct4 by Oct4/Sox2 heterodimer was also shown [15, 16, 17, 18, 19].

Meantime, regulatory links from Nanog to Oct4 and Sox2 appeared to be more complex. Nanog depletion resulted in Oct4 and Sox2 down regulation but Nanog overexpression increased Oct4 level to at least 150% whereas the Sox2 level remained unchanged [14]. Oct4 down regulation in response to Nanog knockdown was confirmed in [20], but Nanog overexpression in these experiments did not increase Oct4 concentration beyond the steady-state level. Furthermore, Nanog expression is low in cells with elevated Oct4 level and high in cells with low Oct4 [20, 21]. In addition, it was reported that Nanog has minimal influence on Oct4 and Sox2 expression [22]. Besides OS activation by Nanog under the question, it was also shown that the main regulator of Nanog transcription is its autorepression. Thus, nowadays, the architecture of the OSN network should be refined to omit Nanog influence on OS expression and incorporate not positive but negative Nanog autoregulation. From this, the OSN gene network becomes a system where one of the genes is repressed by its own product. Understanding of such system behavior is not complete without consideration the transcriptional and translational delays in its functioning [23].

Nanog really plays a special role in OSN triad activity. Nanog overexpression in mESCs maintains pluripotency independently of LIF signaling [24, 25], whereas Oct4 and Sox2 overexpression drives mESCs to mesendodermal and neural ectodermal differentiation, respectively [26, 27, 28]. However, Nanog was not included in the most efficient “cocktail” to induce pluripotency in mouse somatic cells [29] containing Oct4 and Sox2 along with Klf4 and cMyc. Nevertheless, Nanog overexpression accelerates reprogramming of somatic cells to a pluripotent state [30, 31] and activation of the endogenous Nanog and Oct4 is one of the final events in this process [32]. Nanog is necessary for pre-iPSCs (dedifferentiated intermediates) to acquire ground state pluripotency [33]. Whereas Nanog autorepression is mediated by association of Nanog and Zfp281 proteins with NuRD (Nucleosome Remodeling and Deacetylase) complex and Zfp281 inhibits transition of pluripotent iPSCs to ground state by restricting activation of endogenous *Nanog* [34].

Transient up and downregulation of endogenous Nanog was recorded under serum/LIF conditions in individual mESCs predisposing them toward ground stage and differentiation, respectively [35, 36, 37, 38]. Depending on culture conditions the percentage of Nanog-low cells may vary from 5% till 35% [36, 39]. Mathematical modeling on Nanog heterogeneity demonstrated that it might arise from transcriptional noise [36, 40]. Moreover, noise may be sufficient to trigger reactivation of the core pluripotency switch and reprogramming to a pluripotent state [41]. Nevertheless, Wu *et al*. investigated sensitivity and robustness of the Nanog gene network by means of a stochastic model and identified that the system dynamics is sensitive to positive regulation from the Oct4-Sox2 complex [42]. Before Nanog autorepression was shown experimentally by [22], *in silico* simulations demonstrated that Nanog autorepression even indirect provides for sustained oscillations in the system without noise [36, 40]. Comparing sustained oscillations and stochastic fluctuations in an agent-based model suggested that the noise-driven model more consistently explain Nanog expression dynamics in mESC populations. Coupling in simulations the OSN network to extracellular signals provided evidence that stochastic autocrine feedback loops might also generate fluctuations in *Nanog* expression [43]. Meanwhile, a single-molecule fluorescent *in situ* hybridization used to detect *Nanog* mRNA in mESCs, cultured in serum/LIF or 2i/LIF media, offered evidence for the stochastic nature of *Nanog* expression in pluripotent mESCs, independently of the culture conditions implying that NANOG fluctuations are not dependent on autocrine ERK signaling mediated by FGF4 [44].

**Fig 1:**
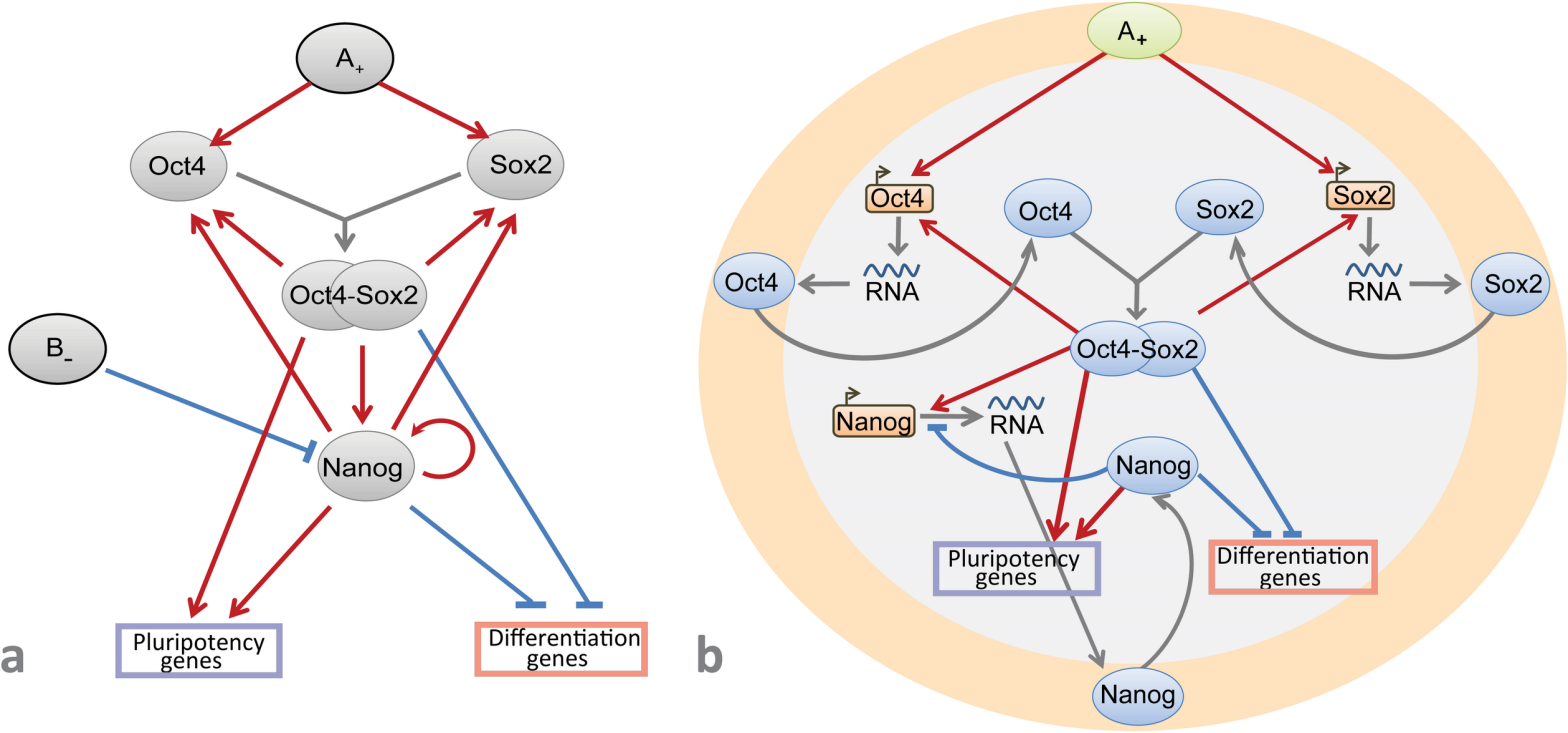
**A:** The core transcriptional network of the factors orchestrating the pluripotency and differentiation genes (suggested by [10]). External A_+_ and B_-_ signals activate and repress expression of *Oct4*, *Sox2* and *Nanog* genes, correspondingly. Oct4 and Sox2 form a heterodimer, Oct4/Sox2, which positively regulates *Oct4*, *Sox2* and *Nanog* expression. Nanog directly induces *Oct4*, *Sox2* and its own expression. Oct4/Sox2 heterodimer and Nanog positively regulate pluripotency genes and repress differentiation genes. **B:** The revised core gene network suggested in this paper, in which transcription and translation processes were added; external signal B- is removed and positive signal A+ activates transcription of *Oct4* и *Sox2* genes. Nanog represses its own transcription and does not influence on *Oct4* and *Sox2* expression.

However, the mutual regulatory role of Nanog autorepression and the strength of the signals inducing *Oct4* and *Sox2* expression in self-renewal and differentiation of mESC as well as an independence/dependence of *Oct4/Sox2* expression from Nanog activation have not been studied *in silico* yet. To sum it up so far, we propose the strength of Oct4 and Sox2 activation and complexity of the Nanog autorepression feedback as key forces governing pluripotency—differentiation switch (Fig 1B).

Herein, we present results on modeling of the mouse ESC core regulatory circuit revisited according to the recent experimental data. The first model describing bistable behavior of the core circuit comprises very simple gene network–only core genes Oct4, Sox2 and Nanog enhancing activity of each other (Fig 1A). Here we revise this model [10] by adding new data on Nanog autoinhibition and taking off Nanog enhancement of Oct4 and Sox2 activity due to controversy records on this topic [22]. As shown in Fig 1B, unlike the initial model we also take into account compartmental localization of each gene transcription in the cell nucleus and maturation of functionally active proteins encoded by core pluripotency genes in the cytoplasm in order to developed model more precisely describes biomolecular events during differentiation and reprogramming processes.

Based on numerical simulations and the parameter continuation method [45] we identified patterns of the ESC states, which existence mainly depends on two following model parameters: the degree of Oct4 and Sox2 activation and molecular complexity of Nanog autorepression. We hypothesize that identified dynamical domains might correspond to naive and primed ES cells and intermediate states between them. To address heterogeneity in Nanog expression, we argue that Nanog autorepression and updated topology of the core network can be internal trigger for oscillations in Nanog expression levels. Eventually, we investigate extended model accounting for positive feedback loops, in which Nanog is an inducer of *Oct4* and *Sox2* transcription. Both model modifications have bistable, switch-like behavior in the same range of parameter values. Moreover, considering the additional positive feedback loops did not result in emergence of the new type of the model behavior but just enhanced the ESC naïve state traits.

It is worth to note that behavior of such complicated system as regulatory machinery of pluripotency\differentiation switch should not be constrained by consideration of only core gene network and extension of transcriptional networks incorporating epigenetic level of pluripotency regulation as well as regulatory circuits mediated via ncRNAs is needed [2,5,9]. However, obtained *in silico* results for the core regulatory circuit can not only provide the explanation and insight into dynamical behavior of the studied biological system, but serve as the basis or starting point to boost follow-up experimental-theoretical investigations.

## Results and discussion

### The model structure

To study regulatory mechanisms of the mESC maintenance and differentiation, the core circuit structure has been revisited. The previously developed model for interaction of core factors in mESC [10] as well as additional experimental data on *Nanog* expression regulation and its interplay with Oct4 and Sox2 [22] served as the basis for the new dynamical model (Fig 1; Materials and Methods). We have also taken into account occurrence of OSN transcription and following translation processes in separate cell compartments (nucleus and cytoplasm). It led to an addition of intracellular mRNA and protein transport simultaneously with the emergence of such parameters as basal transcription rates of the OSN genes, activation or inhibition thresholds, efficiencies of transcription factor binding to a promoter site, as an example. Values of the parameters and corresponding references are represented in the Materials and Methods (Table 1). Degradation rates for OSN mRNAs and proteins were taken from published data [27, 46, 47].

**Table 1:** Biological relevance and values of the model parameters.

Parameter	Value	Biological interpretation	Reference
**a**_**1**_	1	*Oct4* transcription rate	
**a**_**2**_	0.0001	basal transcription level of the *Oct4* gene	10
**a**_**3**_	1	maximal activation constant of *Oct4* transcription at a saturating level of the external signal A	10
**A**	0	signal A+ concentration	10
**a**_**4**_	0.01	maximal activation constant of *Oct4* transcription at a saturating level of the Oct4-Sox2 complex	10
**a**_**5**_	1	effective dissociation constant between Oct4-Sox2 complex and the promoter of *Oct4* gene	
**a**_**6**_	2	Hill coefficient for *Oct4* transcription regulation by Oct4-Sox2 heterodimer	
**a**_**7**_	0.0011	activation constant of *Oct4* transcription at a basal level of the external signal A	10
**a**_**8**_	0.001	activation constant of *Oct4* transcription at a basal level of the Oct4-Sox2 complex	10
**a**_**9**_	0.1	decay rate for the Oct4 mRNA	
**a**_**10**_	1	*Sox2* transcription rate	
**a**_**11**_	0.0001	basal transcription level of the *Sox2* gene	10
**a**_**12**_	1	maximal activation constant of *Sox2* transcription at a saturating level of the external signal A	10
**a**_**13**_	0.01	maximal activation constant of *Sox2* transcription at a saturating level of the Oct4-Sox2 complex	10
**a**_**14**_	1	effective dissociation constant between the Oct4-Sox2 complex and the promoter of *Sox2* gene	
**a**_**15**_	2	Hill coefficient for *Sox2* transcription regulation by the Oct4-Sox2 heterodimer	
**a**_**16**_	0.0011	activation constant of *Sox2* transcription at a basal level of the external signal A	10
**a**_**17**_	0.001	activation constant of *Sox2* transcription at a basal level of the Oct4-Sox2 complex	10
**a**_**18**_	0.1	decay rate for the Sox2 mRNA	27;46–47
**a**_**19**_	0.05	Oct4-Sox2 complex association rate	10
**a**_**20**_	0.5	decay rate for the Oct4-Sox2 heterodimer	
**a**_**21**_	0.001	Oct4-Sox2 complex dissociation rate	
**a**_**22**_	0.0093	transport rate of the Oct4 protein from cytoplasm to nucleus	
**a**_**23**_	0.0081	decay rate for the Oct4 protein in the nucleus	27;46–47
**a**_**24**_	0.5	translation rate of the Oct4 protein	
**a**_**25**_	0.00001	decay rate for the Oct4 protein in the cytoplasm	27;46–47
**a**_**26**_	0.0093	transport rate of the Sox2 protein from cytoplasm to nucleus	
**a**_**27**_	0.0081	decay rate for the Sox2 protein in the nucleus	27;46–47
**a**_**28**_	0.5	translation rate of the Sox2 protein	
**a**_**29**_	0.00001	decay rate for the Sox2 protein in the cytoplasm	27;46–47
**b**_**1**_	1	*Nanog* transcription rate	
**b**_**2**_	1	basal transcription level of the *Nanog* gene	
**b**_**3**_	0.005	maximal activation constant of *Nanog* transcription at a saturating level of the Oct4-Sox2 complex	10
**b**_**4**_	1	effective dissociation constant between the Oct4-Sox2 complex and the promoter of *Nanog* gene	
**b**_**5**_	2	Hill coefficient for *Nanog* transcription regulation by the Oct4-Sox2 heterodimer	
**b**_**6**_	0.001	activation constant of *Nanog* transcription at a basal level of the Oct4-Sox2 complex	10
**b**_**7**_	1	inhibition constant of *Nanog* transcription at a basal level of the Nanog protein	
**b**_**8**_	0.000995	constant of a cooperative effect upon an joint impact of Oct4-Sox2 and Nanog proteins on *Nanog* transcription	10
**b**_**9**_	1	effective dissociation constant between the Nanog protein and the promoter of the *Nanog* gene	
**h**	2	Hill coefficient for Nanog transcription regulation via Nanog autoinhibition mechanism	
**b**_**10**_	0.005	decay rate for the Nanog mRNA	27;46–47
**b**_**11**_	0.0093	transport rate of the Nanog protein from cytoplasm to nucleus	
**b**_**12**_	0.0081	decay rate for the Nanog protein in the nucleus	27;46–47
**b**_**13**_	0.5	translation rate of the Nanog protein	
**b**_**14**_	0.00001	decay rate for the Nanog protein in the cytoplasm	27;46–47
**c**_1_^*^	0.0001	basal transcription level of pluripotency factors	
**c**_**2**_	0.1	maximal activation constant for transcription of pluripotency factors at a saturating level of the Oct4-Sox2 complex	
**c**_**3**_	0.1	constant of a maximal cooperative effect upon an joint impact of Oct4-Sox2 and Nanog proteins on transcription of pluripotency factors	
**c**_**4**_	0.001	activation constant for transcription of pluripotency factors at a basal level of the Oct4-Sox2 complex	
**c**_**5**_	0.01	constant of a cooperative effect upon an joint impact of Oct4-Sox2 and Nanog proteins at their basal level on transcription of pluripotency factors	
**c**_**6**_	0.05	decay rate for pluripotency factors	
**c**_7_^*^	1	basal transcription level of differentiation factors	
**c**_**8**_	0.001	inhibition constant for transcription of differentiation factors at a basal level of the Oct4-Sox2 complex	
**c**_**9**_	0.01	constant of a cooperative inhibitory effect upon an joint impact of Oct4-Sox2 and Nanog proteins at their basal level on transcription of differentiation factors	
**c**_**10**_	0.01	decay rate for differentiation factors	
**q**_**1**_	0.01	maximal activation constant of *Oct4* transcription at a saturating level of the Nanog protein	
**q**_**2**_	0.001	activation constant of *Oct4* transcription at a basal level of the Nanog protein	
**q**_**3**_	0.01	maximal activation constant of *Sox2* transcription at a saturating level of the Nanog protein	
**q**_**4**_	0.001	activation constant of *Sox2* transcription at a basal level of the Nanog protein	
**p**_**1**_	1	effective dissociation constant between the Nanog protein and the promoter of the *Oct4* gene	
**h**_**1**_	2	Hill coefficient for *Oct4* transcription regulation by the Nanog protein	
**p**_**2**_	1	effective dissociation constant between the Nanog protein and the promoter of the *Sox2* gene	
**h**_**2**_	2	Hill coefficient for *Sox2* transcription regulation by the Nanog protein	

* assumption that a transcription rate for the factor is the constant and equals to 1.

With the updates to the core network above, we added a set of differential equations (Materials and Methods) using generalized Hill functions [48]. The generalized Hill functions allow to elaborate both mathematical model construction and quantitative description of biological systems with partially known or poorly understood molecular mechanisms. For constructing the diagram of stationary solutions for the OSN network we applied a software package STEP+ [49] based on parameter continuation method [45, 50]. Using STEP+ we analyzed influence of parameter values on the model solutions and their stability. The diagrams of stationary solutions combined with stability characteristics and obtained by this way describe the dynamics or behavior of the autonomous system, its states and transitions among them. In particular, a multiplicity of stationary solutions coexisting in a range of the parameter values mean that the autonomous system may have a hysteresis phenomenon. If a single unstable stationary solution only exists in some range of the parameter values, it argues for self-oscillations occurrence. Below we describe in detail the whole set of stationary solutions coming from analysis of our model using this tool.

### Threshold in OS activation defining differentiation-pluripotency transition

**Fig 2:**
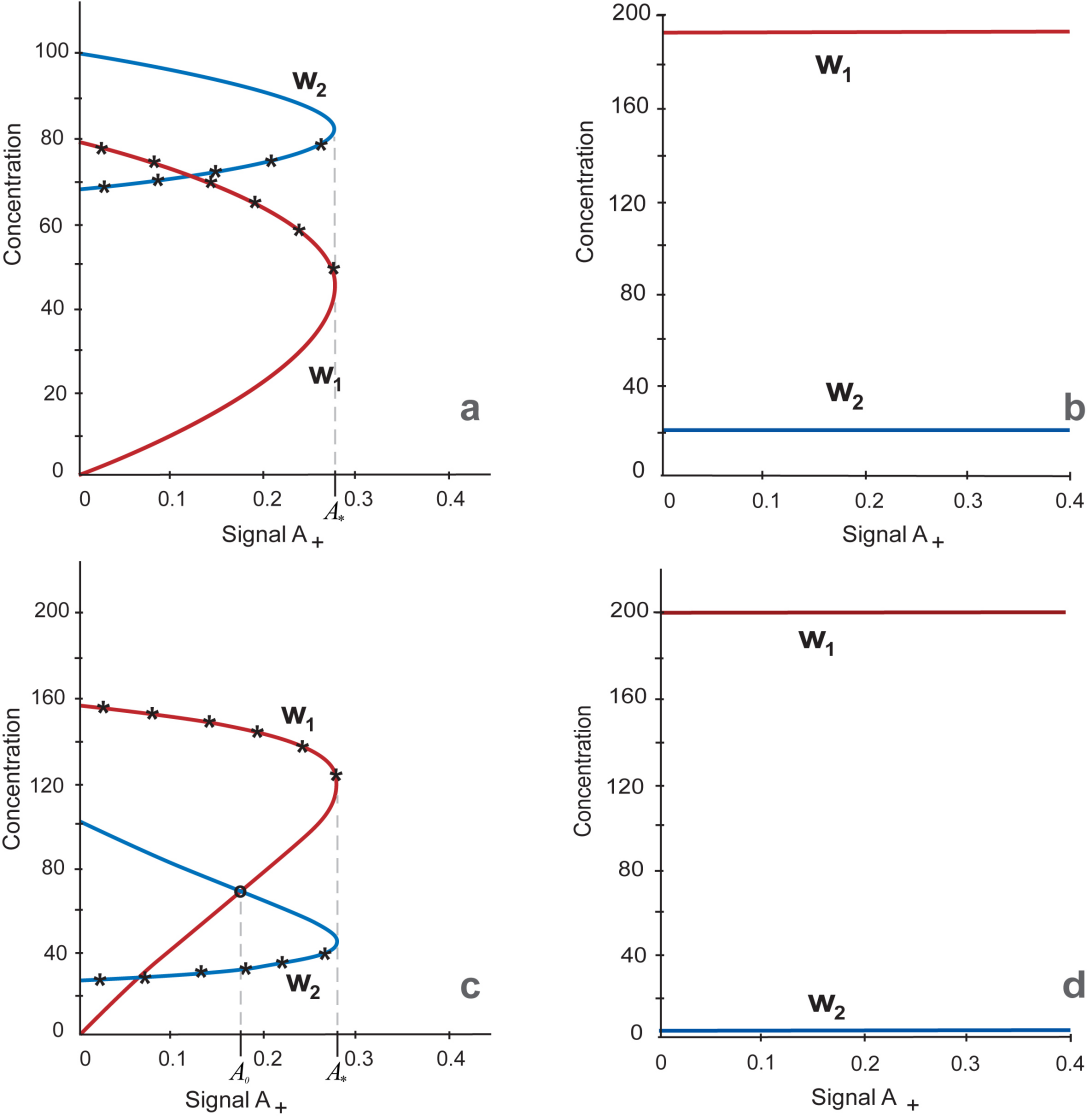
Multiplicity of stationary solutions representing as *w*_*1*_ and *w*_*2*_ dependence on the parameter *A*, *0* ≤ *A* ≤ *0*.*4* at *h = 6* (2a and 2b) and *h = 2* (2c and 2d). a. Initial steady state values *w*_*2*_>*w*_*1*_ and *A = 0* simulate differentiation state. The *w*_*1*_*/w*_*2*_ ratio while A is growing upon *A* = A_*_ = *0*.*277* (the turning point), corresponds to the differentiation steady state. Asterisks indicate arcs of the graphs with unstable solutions. b. Initial steady state values *w*_*1*_> *w*_*2*_ and *A* ˃ A_*_ simulate the pluripotent state. The graph of the steady state while decreasing A upon *A ≥ 0* (including the range *0* ≤ *A* ˂ A_*_) is depicted. Both Fig 2A and 2B show that three states (two stable and one unstable) exist in the range 0 ≤ *A* ˂ A_*_ *= 0*.*277*, while there is one steady state, when *A* ˃ A_*_. c. Stationary solution with initial steady state values *w*_*2*_>*w*_*1*_ and A = 0 corresponding to the differentiated cell. *w*_*1*_ and *w*_*2*_ variables have turning points at *A* = A_*_. Asterisks indicate unstable solutions. d. Stationary solution with initial steady state values corresponding to the pluripotent cell. The Fig 2C and 2D shows that three states (two are stables and one is unstable) exist in the range *0* ≤ *A* ˂ A_*_ *= 0*.*277*, while there is one steady state, when *A* ˃ A_*_ and this is the pluripotent state only.

OSN targets are subdivided into pluripotency target genes (PTGs) and differentiation target genes (DTGs) encoding for pluripotency and differentiation factors, respectively. To define a cell state, we considered the ratio between values of *w*_*1*_ and *w*_*2*_ denoting concentrations of proteins, encoding by PTGs and DTGs, respectively, at a stable steady state. If *w*_*1*_ is considerably more than *w*_*2*_, the OSN gene network is in pluripotency state and if the ratio is opposite, it is in differentiation state. It is notable that unstable solution indicated by asterisks in Fig 2 and further, cannot be used for cell state determination and is referred only as unstable. The induction of pluripotency that corresponds to the switch from *w*_*1*_ < *w*_*2*_ to *w*_*1*_ > *w*_*2*_ is caused by A signal activating *Oct4* and *Sox2*. The latter simulates either OS ectopic expression via transgenic factors or their activation by any other factors inducing pluripotency. The value of the parameter A equals to zero in differentiated cells before induction of pluripotency and during this process we increase it till 0.4. To study outcomes of above-mentioned structural reorganization of the core circuit on the model behavior we traced values of *w*_*1*_ and *w*_*2*_ variables upon variation of two model parameters: *h*, which is a Hill coefficient determining the nonlinear nature for Nanog negative regulation of *Nanog* expression and *A*_,_ the parameter determining a value of the signal A activating *Oct4* and *Sox2* expression. To consider a high-level complexity of *Nanog* autorepression, we initially constructed curves of *w*_*1*_ and *w*_*2*_ dependence on *A* values varying from 0 till 0.4 and the parameter *h* equaled to 6.

As it follows from Fig 2A (initial condition—differentiated cells) and Fig 2B (initial condition—pluripotent cells), there are two following types of the steady state’s dependencies on the parameter *A*, (i) stable differentiation, the steady state in the range 0 ≤ A ˂ A_*_ *= 0*.*277* with turning point upon *A* = A_*_ (Fig 2A) and (ii) stable pluripotency, the steady state at all *A ≥ 0*, including the range 0 ≤ A ˂ A_*_ (Fig 2B). Thus, depending on initial steady state values and at *h = 6* generally there are three states in the range 0 ≤ A ˂ A_*_, two steady states (pluripotency and differentiation) and one unstable state (differentiation/pluripotency), two of them starting from differentiated (Fig 2A) and one from pluripotent cells (Fig 2B). Whereas only one state exists when *A* ˃ A_*_ and this is the steady state from pluripotent condition. The existence of the unstable states (Fig 2A) coincide with the existence of the early phase of stochastic gene expression during induction of pluripotency [51].

If the parameter *A* value is higher than the threshold value A_*_ *= 0*.*277*, pluripotency state was observed independently on initial values of the model variables. This simulation confirms experimental observations that once ground pluripotency is established, reduced Oct4 level in cells growing on 2i/LIF medium did not lead to loss of the pluripotent state [21, 52]. These Oct4-low cells have nondynamic unimodal Nanog level [21] simulated in our model by the fixed value of the Hill coefficient for *Nanog* autorepression (*h = 6*). To consider the system behavior in more wide range of the *h* parameter we investigated the stability of stationary solutions under different values of the Hill coefficient for *Nanog* autorepression.

### The strength of Oct4 and Sox2 activation and non-linearity of Nanog autorepression determine the choice between pluripotency and differentiation states and their stability

The investigation of the system stability showed that there is a domain defined by *h* values above threshold, h_*_ = 8.68, where system behavior is practically independent from *A* value *A ≥ 0*. If we consider the range *A ≥ 0*, h > h_*_ all states of the system will be unstable and represented only by an oscillatory mode.

**Fig 3:**
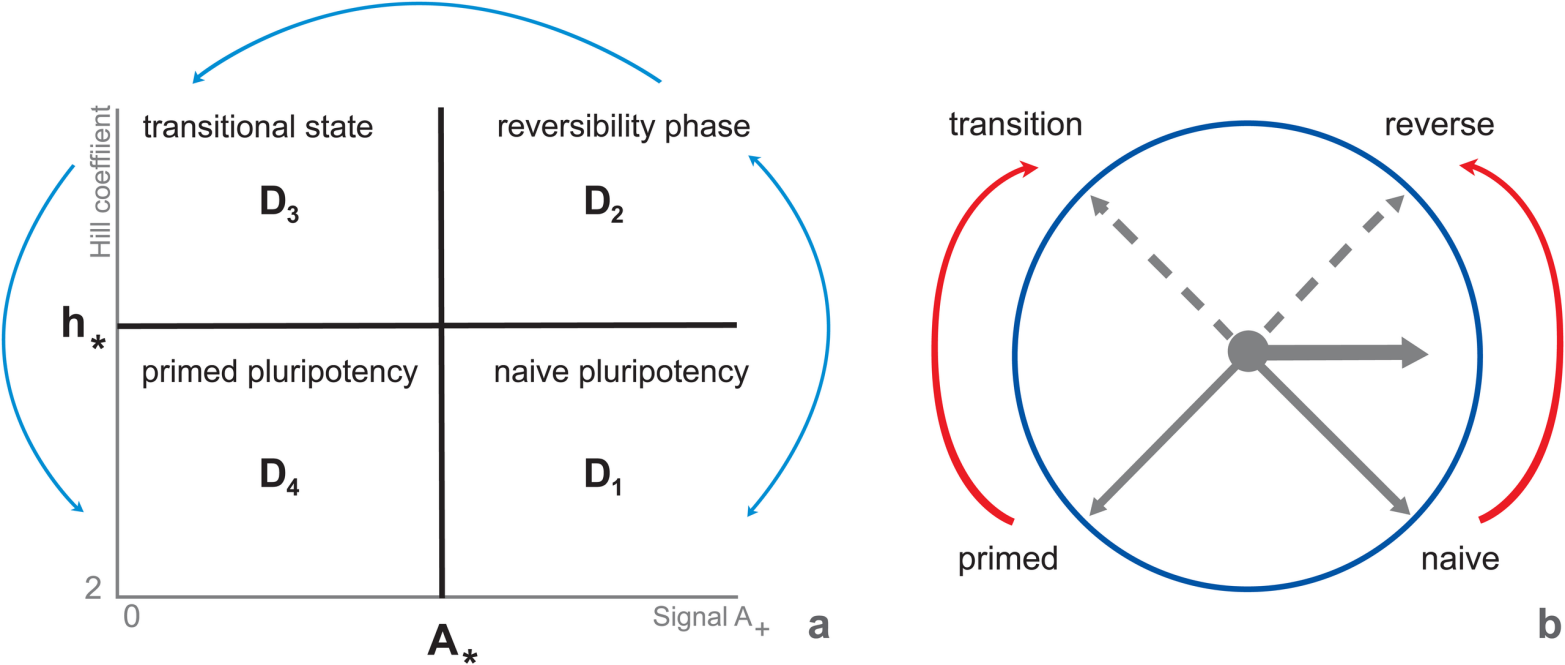
Multiplicity and stability of stationary solutions depending on parameters *A* and *h*. *D*_*4*_ domain comprises a single stable steady state, pluripotency; *D*_*2*_domain encompasses a single unstable state (oscillation). *D*_*3*_ domain includes three unstable states (oscillations); *D*_*4*_ domain contains three states, from which two (pluripotency and differentiation) are stable and one (transition between these states is unstable (according to Fig 2A). Domains (a) predicted by the model and (b) their correspondence to developmental progression of ESCs from the naïve pluripotency (the ground state) to lineage commitment according to [61]. The initial phase of exit from the ground state is asynchronous in the cell population and reversible until the complete dissipation of naïve state factors (reviewed in [59, 61, 62]). Cells reaching a transitional point after 2i withdrawal are competent for lineage specification and characterized by the absence of both groups, naïve factors and lineage markers. The late phase of pluripotency (primed pluripotency) is characterized by nascent expression of lineage specification factors. The “clock model” was proposed as a route of consistent transitions with the dual mechanism of hour hand movement depending on the initial cell state: pluripotent (counter-clockwise movement of black solid arrows) or differentiated (clockwise movement of black solid arrows). Red arrows, in turn, reflects directions from naïve to reverse-transition-primed stages (developmental progression during differentiation) or from primed to transition-reverse-naïve states (developmental progression during reprogramming into pluripotent state), while dotted black arrows were added to underline intermediate reverse and transitional states to which domains *D*_*2*_ and *D*_*3*_ correspond, respectively.

Describing the dependence of steady state stability on parameters *A* and *h*, we found four following domains on the plane (*A*, *h)* separated by the lines A = A_*_ *= 0*.*277* and h = h_*_ *= 8*.*68* (Fig 3):

$${\text{D}}_{1}=(\text{A}>{\text{A}}_{*},\;2<\text{h}<{\text{h}}_{*}),\;{\text{D}}_{2}=(\text{A}>{\text{A}}_{*},\;\text{h}>{\text{h}}_{*}),\;{\text{D}}_{3}=(0\leq \text{A}<{\text{A}}_{*},\;\text{h}>{\text{h}}_{*}),\;{\text{D}}_{4}=(0\leq \text{A}<{\text{A}}_{*},\;2<\text{h}<{\text{h}}_{*})$$

Note that the minimal value of the Hill coefficient, which equals 2, is explained by the fact that Nanog homodimer is the core protein complex in ESCs [53, 54]. It was experimentally demonstrated, that Nanog protein dimerization is vital for stem cell self-renewal and pluripotency. Higher level of the parameter value can be interpreted by means of accounting for larger protein complexes with Nanog participation and/or extended gene regulatory circuits for negative feedbacks regulating *Nanog* expression [55, 56, 57]. As can be seen from Fig 2, *D*_*4*_ domain contains three states. The stationary solution diagrams of these states reproduce analogous diagrams and stability properties represented in Fig 2. It means that two solutions in the domain are stable and correspond to differentiation and pluripotency, while the third solution is unstable and tend to the differentiation according to the model analysis (Fig 2A). There is the single and stable stationary solution in *D*_*1*_ domain, which corresponds to the pluripotent cell and illustrated at *h = 6*, Fig 2B. Stationary solutions in *D*_*2*_ and *D*_*3*_ domains comply with the stationary solutions in *D*_*1*_ and *D*_*4*_ domains, correspondingly, but the qualitative difference is that all stationary solutions are unstable in *D*_*2*_ and *D*_*3*_ domains. Model solutions in *D*_*2*_ and *D*_*3*_ domains exhibit oscillations for any initial conditions. Altogether *D*_*2*_ and *D*_*3*_ domains may constitute the formative pluripotency suggested by Smith (2017) as intermediate phase between naïve and primed pluripotency [1]. Thus, the formative phase might be associated with increased complexity of Nanog repression simulated via *h* in our model. Coinciding with this Nanog is absent from immediate post-implantation epiblast [58, 59] corresponding to the formative phase of ESCs [1], Nanog downregulation is necessary for initiation of lineage specification [27, 35] and Nanog overexpressing ESCs resist differentiation to EpiSCs [60].

It is noteworthy that the number of domains and their properties match to observed ESC developmental progression from naive to primed pluripotency (reviewed in [61]). It was also demonstrated that both bFGF/Activin A and L-proline induce naïve to prime transition, but after L-proline treatment ESCs mainly reached a fully reversible early phase of this transition [62]. Naïve to prime transition is reversible until complete dissipation of ground state factors occurs [59, 61]. This denotes reaching the transitional stage at which neither ground state factors (e.g. Nanog, Essrb and Tcp3l1) not lineage markers (e.g. Bry, Sox17 and Brn2) are expressed, whereas Oct4 and Sox2 are present. Cells at the transitional stage become competent for lineage specification. Nanog is high at the ESC ground state [21, 59]. Nanog downregulation is necessary and sufficient for acquiring competence for lineage specification [27, 35]. Exit from the ground state occurs asynchronously in individual cells [62].

Revealed *in silico* boundaries of the parameter values that constrain functional ESC states and transitions between them, in turn, can be represented by “clock model” with the dual mechanism of hour hand movement depending on the initial cell state: pluripotent or differentiated and the road map depending on *h* value (Fig 3A and 3B). At *h = 6* corresponding approximately intermediate Nanog level (Fig 2B) we fail to reach differentiation state by decreasing Oct4 and Sox2. This again emphasizes the key role of Nanog downregulation in differentiation suggesting its occurrence via increasing complexity (nonlinearity) of Nanog negative direct and indirect feedbacks.

After increase in the Nanog level by decreasing its downregulation feedbacks, the resulting diagrams of stationary solutions at *h = 2* (Fig 2C) differ from the diagrams in Fig 2A built with *h = 6*. Stable arcs of curves for *w*_*1*_ and *w*_*2*_ variables, belonging to the first type of stationary solution, have intersection point to the left of the turning point (Fig 2C). The intersection occurred at the *A = A*_*0*_ *= 0*.*173*. Since *A* value of intersection point, *A*_*0*_ *= 0*.*173* at *h = 2* is less than the value of turning point, A_*_ *= 0*.*277* at *h = 6*, the pluripotent state of cells existing in *D*_*1*_ domain continues to *D*_*4*_ domain at *A*_*0*_ < A ≤ A_*_. However, the differentiation state resides in *D*_*4*_ domain only at *0 ≤ A ≤ A*_*0*_.

Theunissen and Silva (2012) suggested that the key role of high endogenous Nanog in reprogramming might closely resembles its role in establishment of the naive pluripotent epiblast in early mouse embryogenesis [63]. At least initially, a weak expression of Nanog is observed and its level is variable between blastomers [64], which correspond to *D*_*2*_ and *D*_*3*_ domains in our diagrams. This is followed by general upregulation of Nanog expression with further differentiation of primitive endoderm and preimplantation epiblast. The latter one corresponds to the ground ESC state (reviewed in [65]).

**Fig 4:**
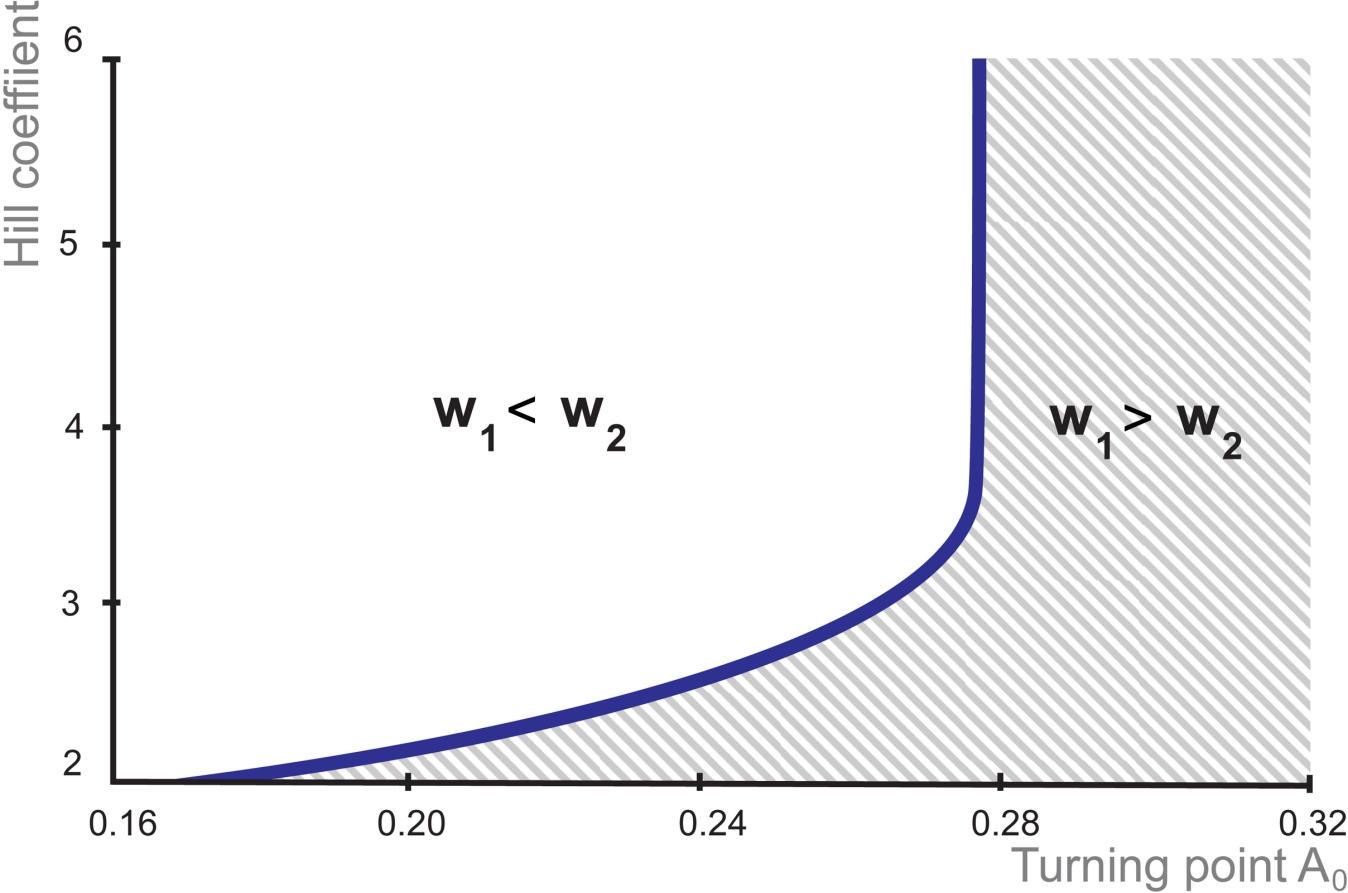
Dependence of the Hill coefficient value on the value of OS activating signal at which transition from differentiation to pluripotency occurs. The horizontal and vertical axes represent the values of parameters *A*_*0*_ and *h*, respectively. The curve distinguishes between pluripotency and differentiation states.

Moreover, the graph in Fig 4 indicates that intersection point *A*_*0*_ *= 0*.*173* at *h = 2* raises within the bounds *0*.*16 ≤ A*_*0*_ *< 0*.*277* simultaneously with the increase of the parameter *h* from 2 until 3.5. But starting from *h* = 3.5 and higher *A*_*0*_ value keeps constant and equals to *A*_*0*_ = A_*_ *= 0*.*277* making in this area the boundary condition between differentiation and pluripotent states not dependent on non-linearity (complexity) of the Nanog negative feedback.

Whereas in the region of weak activation of OS genes and low values of the Hill coefficient, which reflects low nonlinear effect of Nanog on its own expression, the dependency curve grows gradually. Thus, a weak activation of OS genes and low values of the Hill coefficient is sufficient to pre-iPSC/iPSC transition upon the pluripotency induction, while a high-order nonlinearity of *Nanog* repression (more complex, involving more number of regulatory interactions and factors) needs the higher value of *OS* expression. Increase in OS expression is limited and at the highest value is independent on values of Hill coefficient (Fig 4).

This simulation fits several experimental observations. Reprogramming occurs gradually and increase in endogenous expression of core genes also occurs gradually starting from very low doses [32, 66]. The observation corresponds to the graph part, where value of the Hill coefficient increases simultaneously with *A*_*0*_ value. Further increase of *h* parameter results in repression of *Nanog* level after a certain threshold, while an activation of *Oct4* and *Sox2* transcription reaches the own threshold. It is known, that ESC self-renewal requires Oct4 and Sox2 maintaining within narrow limits exceeding which lead to ESC differentiation [26, 67]. Our model confirmed the existence of these thresholds and showed that during reprogramming increase in OS levels is accompanied by increase in complexity of Nanog autoregulation. Whereas on serum/LIF media Nanog-positive and Nanog-negative mESCs were recorded, under 2i/LIF conditions where main mESC signaling via MAPK/ERK1/2 and GSK3 is strongly inhibited, the mESCs uniformly express pluripotency markers including Nanog [68, 69]. Nanog autoinhibition persists in 2i/LIF [22], but Nanog-negative cells were not recorded. It is obvious that signaling pathways in serum/LIF are more complex than in 2i/LIF. It allows to suggest that regulation of Nanog autoinhibition in serum/LIF is more complex and order of the nonlinearity is higher. Strikingly, the upper boundary of the parameter range (*h = 6*) quantitatively corresponds the value used in Miyamoto *et al*. (2015) study, in which they examined the gene expression dynamics model with epigenetic feedback regulation to show that differentiation with the loss of pluripotency progresses from the embryonic stem cell state with oscillatory expression of pluripotent genes [70]. Apparently, the high value of Hill parameter in both models reflects the complex structural organization of *Nanog* expression regulation.

Decreasing Oct4 to the level present in Oct4+/- mESC shifts Nanog to the higher level detected in wild type mESCs due to reducing the proportion of Nanog-low or negative cells [21]. The increase in Oct4 level results in establishing of Nanog heterogeneity, in other words increase in Oct4 activation is accompanied by increase in non-linearity of Nanog autoinhibition. The 2i/LIF conditions may correspond to pluripotent stem cells from the lower part of the curve in Fig 4, whereas serum/LIF corresponds to its upper part. In other words, in mouse ES cells under 2i/LIF conditions the values of both *A*_*0*_ and *h* are lower than in serum/LIF medium. It was also experimentally found that using 2i/LIF media at transition from pre-iPSCs to iPSCs is more preferable than serum/LIF due to increase in the efficiency of reprogramming [71].

### Nanog expression heterogeneity in *D*_*2*_ and *D*_*3*_ domains

In the proposed model the parameter *A* characterizes OS activation by the signal A. *OS* expression dynamics linearly depends on the *A* value, while dynamics of *Nanog* expression is not so intuitive clear.

**Fig 5:**
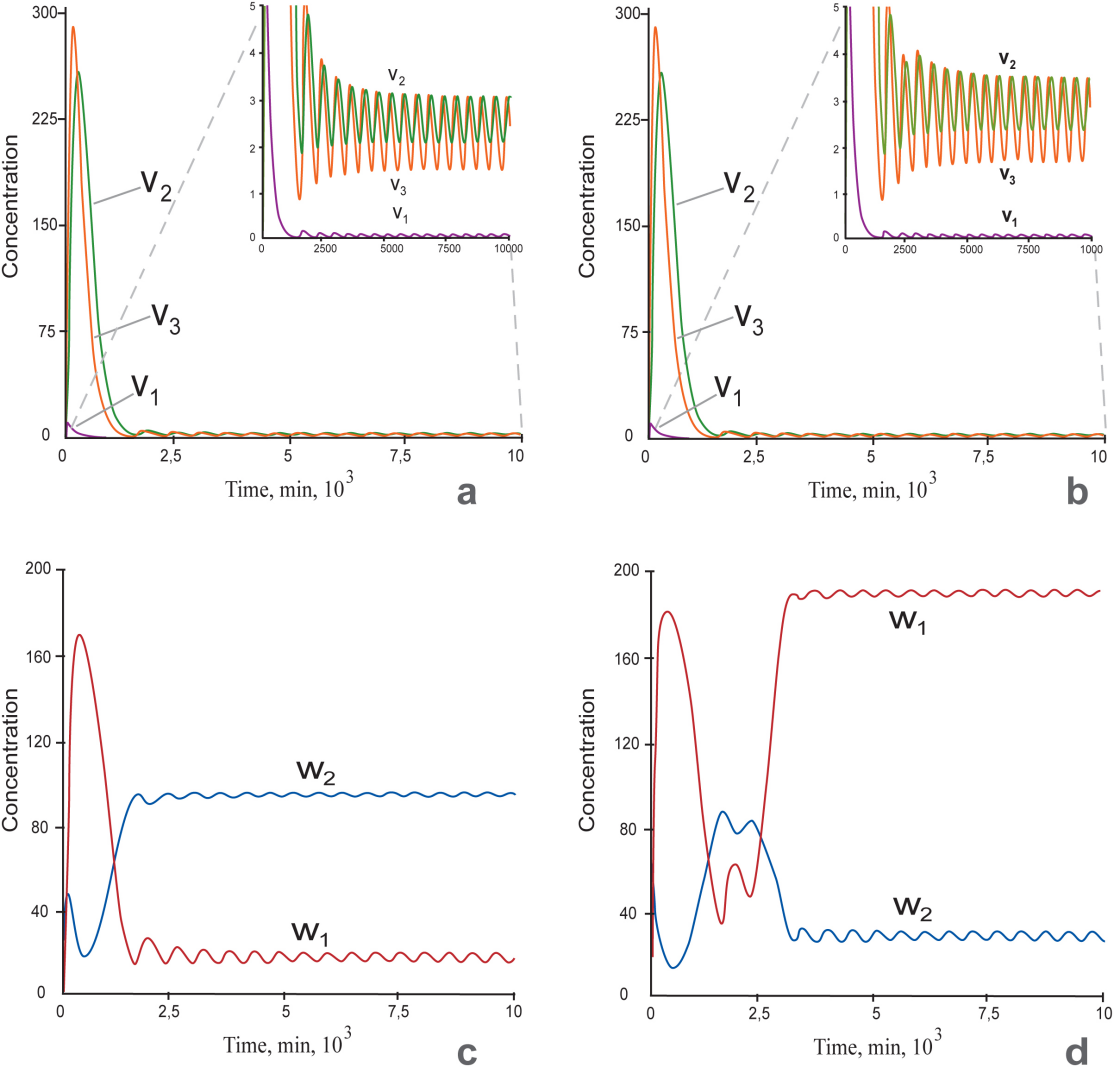
**a-b:** Time series of mRNA and protein expressions for *Nanog* at *h = 10*: *v*_*1*_—*Nanog* mRNA concentration, *v*_*2*_ –Nanog protein concentration in the nucleus, *v*_*3*_—Nanog protein concentration in the cytoplasm; The insets in Fig 5A and 5B represent the same curves as on the main part, but with a zoomed scale of the y-axis. **c-d:** Time series for concentrations of pluripotent (*w*_*1*_) and differentiation (*w*_*2*_) factors. Concentration oscillations of Nanog and pluripotent/differentiation factors occurred at *A = 0*.*2* (a, c) and *A = 0*.*3* (b, d). The other parameters were fixed. c: The pluripotent factors *w*_*1*_ were suppressed and the differentiation factors *w*_*2*_ were expressed. This state corresponds to differentiation. d: Pluripotent factors were highly expressed, and differentiation factors were suppressed. This state corresponds to pluripotency.

Expression of Nanog and some other genes including Nanog targets is heterogeneous on serum/LIF media [35, 36, 68, 69, 72, 73; 74, 75]. The heterogeneity is dynamically maintained, with individual cells exhibiting transient changes in expression levels. *Nanog* heterogeneity has been widely studied by mathematical modeling approach and it was shown that the phenomenon may be inferred from properties of OSN interaction circuit, activity of signal transduction pathways or transcriptional stochasticity induced transitions [12, 36, 40, 43, 76]. Our model also describes this behavior in domains *D*_*2*_ and *D*_*3*_, in which all revealed states are unstable (Fig 3A). In particularly, both *Nanog* mRNA concentration and protein levels in nuclear and cytoplasmic compartments have oscillation dynamics (Fig 5).

The emergence of these attractors occurs at *h > 8*.*68* and as shown in Fig 5A and 5B, both Nanog and pluripotent/differentiation factors (Fig 5C and 5D) have expression oscillations and it does not depend on the level of OS activation (Fig 5A and 5C: *A < 0*.*277*, Fig 5B and 5D: *A > 0*.*277*). In dynamics of pluripotency (*w*_*1*_) and differentiation (*w*_*2*_) factors *A* values influence the proneness of the whole system to differentiation or pluripotency: at *A < 0*.*277*, it biased to differentiation and at *A > 0*.*277* it has pluripotent tendencies.

Negative feedback is a general requirement for oscillatory behavior [77]. For Nanog regulation, the shortest feedback is Nanog autoinhibition. This may have different number of intermediate steps due to involvement of histone acetylation [78], histone methylation [79], DNA methylation [80, 81] or additional regulators [55]. The diversity of additional steps in Nanog autorepression can explain diverse unstable states represented by oscillations in the domain *D*_*3*_.

Therefore, we suppose that obtained unstable states may represent different routes to and types of pre-iPSC states or other incomplete reprogramming derivates. At least oscillations in expression were indicated by real-time imaging for several differentiation genes in partly differentiated cells (for example, for neural progenitors) where these oscillations serve for maintenance of multipotency before the sustained expression of these genes in their lineages after cell fate decisions [82, 83]. Whereas sustained expression of the key differentiation gene in the lineage is the trait of the lineage differentiation steps, simultaneous oscillation expression of the key differentiation genes of several lineages marks the multipotent state of the common proliferating progenitor. Thus, during developmental transitions, the oscillatory expression of several fate determination factors were recorded at the multipotent state, whereas after choosing cell fate only one of them was sustainably expressed in the course of chosen differentiation. Fluctuations and oscillations in gene expression were suggested as a basic character of stemness, potentiality both to proliferate and differentiate [84]. While a loss of oscillatory dynamics leads to differentiation and the loss of stemness, reactivating of oscillatory expression of several key lineage specific genes was predicted to restore pluripotency. It was also shown that counteracting lineage specifiers synergistically induced pluripotency in the absence of both Oct4 and Sox2 [85, 86]. The malignant transformation also looks like differentiation-stemness transition. We can only speculate about the role of increase in non-linearity of Nanog autorepression in this process. Nevertheless, Zfp281 mediates Nanog autorepression by means of the NuRD complex recruitment and inhibits somatic cell reprogramming by repressing Nanog activity [34]. The ZNF281 increased expression played an important role in tumor cell formation and this ZNF281 function was only recently discussed and reviewed [87]. Thus, we hypothesize that in our simulations the border between *D*_*3*_ and *D*_*4*_ domains may describe some experimentally observed transitions from one steady state to another (differentiation-pluripotency or pluripotency-differentiation). In the next section we shall consider in details conditions for this bistability.

### Conditions for differentiation/pluripotency bistability in the *D*_*4*_ domain

As we pointed out in the previous section, the oscillations of differentiation factors were recorded at the multipotency state before differentiation [34, 83]. In mESC cultivated on 2i media where differentiation driving signals (MAPK/ERK1/2 and GSK3) are inhibited, there is uniform expression of pluripotency genes and low to absent expression of differentiation genes [68, 69]. Whereas on LIF/serum media mESCs have fluctuations in expression of Nanog, *Pecam1*, *Rex1(Zfp42)*, *Dppa3 (Stella)*, *Tbx3*, *Klf4*, *Esrrb Tcl1*, *Fgf5*, *Bry* and *Dnmt3b* [35, 36, 68, 69, 72, 73, 74, 88]. There are both pluripotency and lineage specific genes among them. Due to this, mESC on LIF/serum media are more prone to differentiation than those on 2i/LIF [68, 69]. Between naïve pluripotency and differentiation states there is a transition stage (Fig 3A; [61]) with fluctuations in expression of both pluripotency and differentiation genes.

**Fig 6:**
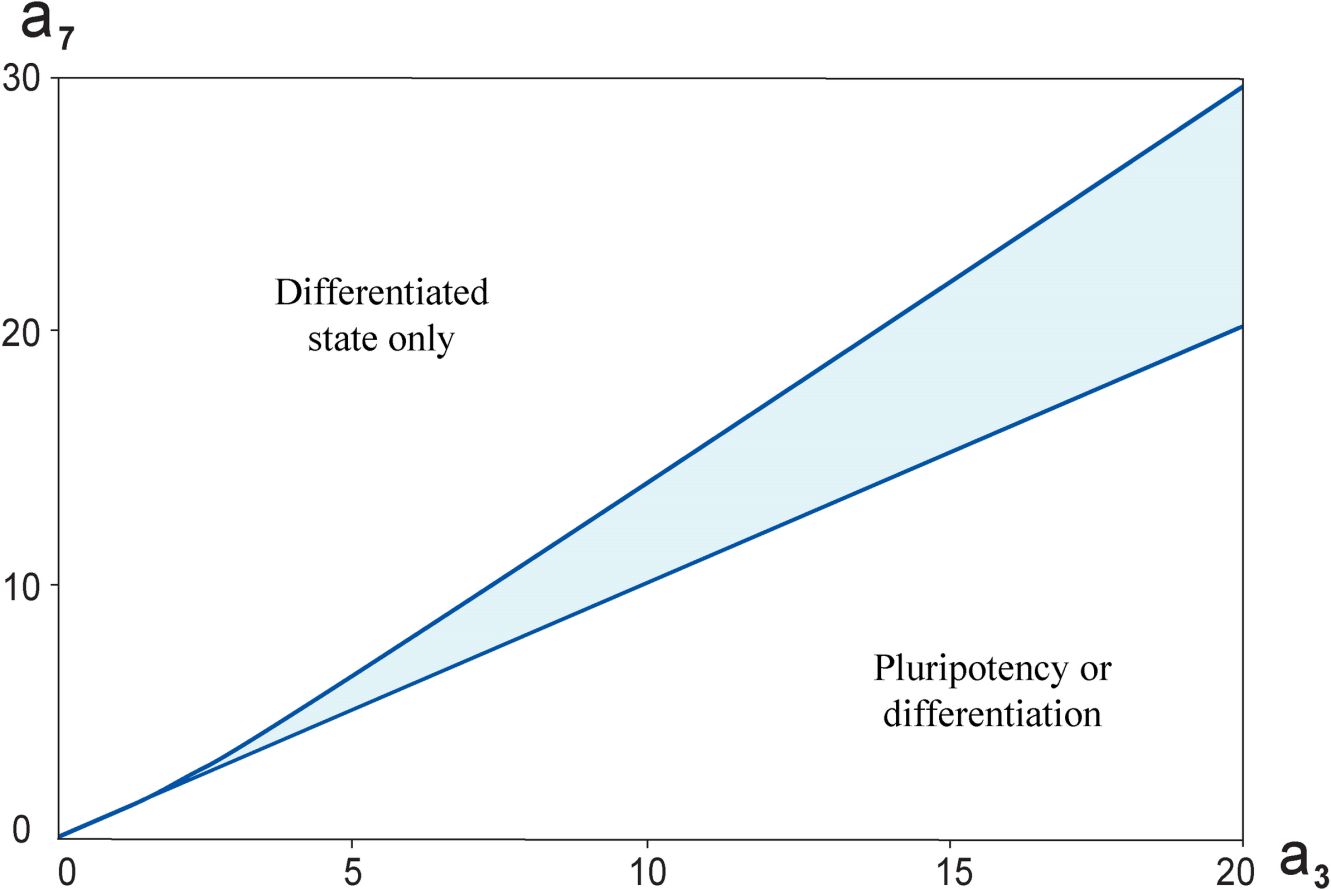
The bistable switch in the core network depending on (*a*_*3*_, *a*_*7*_) parameters and at *h = 6*. Highlighted region is the range of parameter values, having which the system has switch-like behavior. Furthermore, the analysis indicated that a straight line *a*_*3*_ = *a*_*7*_ divides the plane (*a*_*3*_, *a*_*7*_) it into two areas. When *a*_*3*_ < *a*_*7*_, the cell has differentiated state at all values *A* ≥ *0*. When *a*_*3*_ > *a*_*7*_, there will be some *A*_*0*_, that upon *A* > *A*_*0*_ the cell is pluripotent, while at *A* < *A*_*0*_ the cell will differentiate.

**Fig 7:**
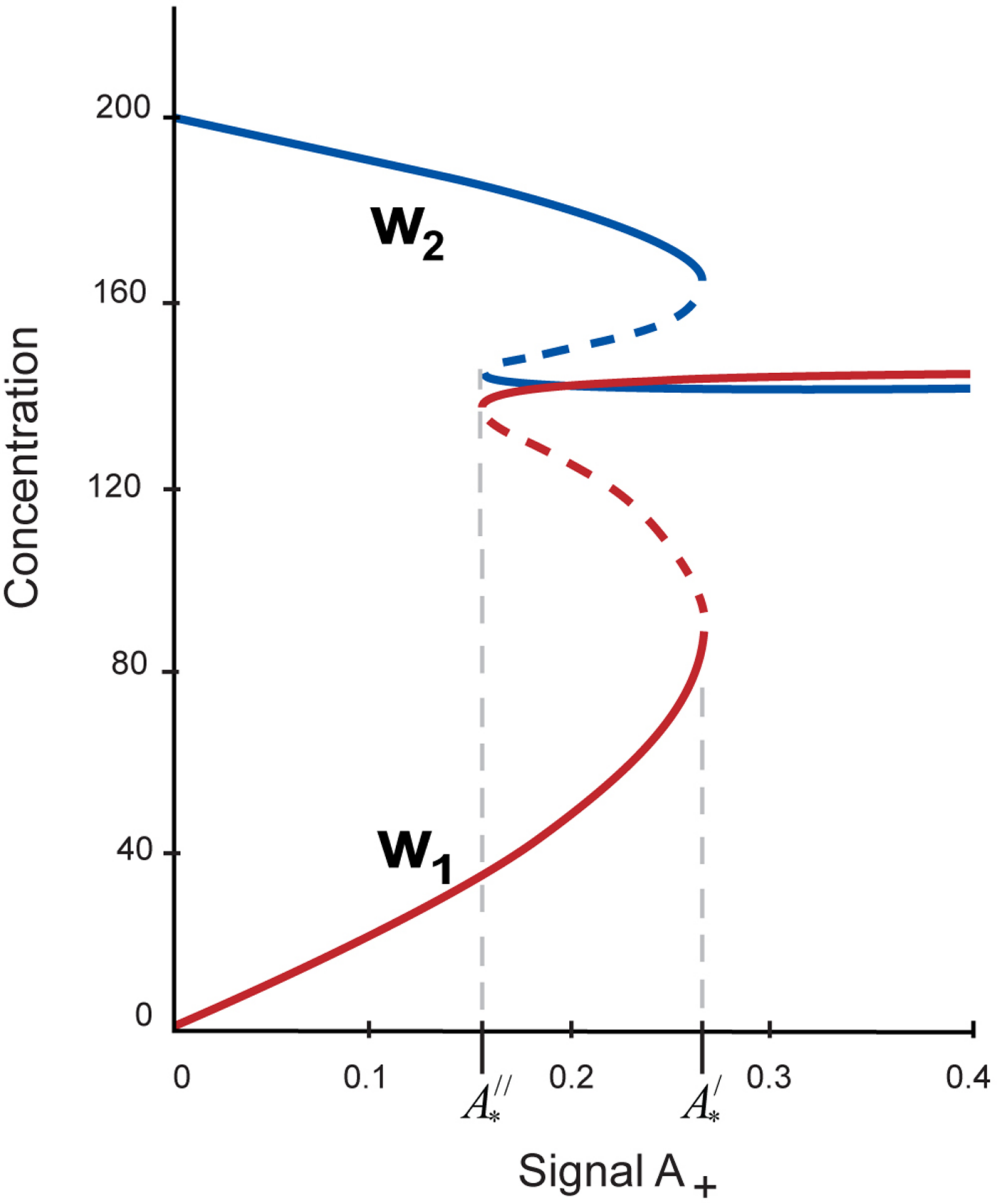
Steady state behavior of the PTGs and DTGs as functions of the parameter *A* upon *h = 6*, *a*_*3*_ *= 14*.*5*, *a*_*7*_ *= 14*. Abscissas of the turning points of *w*_*1*_ and *w*_*2*_ curves with values $A_{*}^{||}=0.1531$ and $A_{*}^{|}=0.2653$ determine the limits of the bistable behavior (marked by asterisks). As the parameter *A* is boosted beyond $A_{*}^{|}=0.2653$, the core network switches to the pluripotent state, while when the external signal is dropped below $A_{*}^{||}=0.1531$, the system switches to differentiation state.

To find out model stationary solutions for pluriponency/differentiation and differentiation/pluripotency transitions with pronounced transition zone we investigated how the model parameters determine the dynamics of the PTGs and DTGs. More precisely, we studied the cell state dependence on parameters, *a*_*3*_ and *a*_*7*_, related to the free energies of A signaling molecule binding to the promotor regions of regulated genes in terms of the statistical mechanics approach [10]_._ The analysis demonstrated that there is a range of the parameters *a*_*3*_ and *a*_*7*_, (Fig 6) for which the system has a bistable switch-like behavior (Fig 7).

As can be seen from Fig 6, one observes the parameters range marked by blue color in which the switch-like behavior is demonstrated. It was numerically found that a straight line *a*_*3*_ = *a*_*7*_ in the plane (*a*_*3*_, *a*_*7*_) splits the wedge-shaped region into two areas: the first one is an approximate boundary between *a*_*3*_ < *a*_*7*_, in which the differentiation state only exists at *A* ≥ *0*, and the second one is an area *a*_*3*_ > *a*_*7*_, in which both pluripotency and differentiation criteria can be performed upon a certain value of the external A signal.

In Fig 7, we show the example of steady state concentrations of *w*_*1*_, PTGs and *w*_*2*_, DTGs depending on the parameter *A*, depicted at *h = 6*, *a*_*3*_ *= 14*.*5*, *a*_*7*_ *= 14***.** There are two turning points ($A_{*}^{||}=0.1531$ and $A_{*}^{|}=0.2653$) defining the hysteresis curve.

The steady state behavior of the pluripotent and differentiation factors encoding by pluripotency (*w*_*1*_) and differentiation (*w*_*2*_) genes are the following:

- With increase of the parameter *A* the beginning of a switch from differentiated to pluripotent state (transit zone) takes place if $A_{*}^{|}=0.2653$;
- With decrease of this external signal, i.e. under differentiation of ESCs, the transition from pluripotency to differentiation occurs at significantly lower value of the signal, $A_{*}^{||}=0.1531$.

The observed model predictions reproduce reprogramming events during both induction of pluripotency and ESC differentiation. The high initial activation of exogenous OS provided by ectopic expression of the transgene OS factors is an essential upon pluripotency induction, followed by preiPSC/iPSC stabilization that is initiated from very low transcription level of the endogenous OS (reviewed in [6]).

During ESC differentiation, the dynamics is more complicated: Oct4 level increased but Sox2 level was repressed at initiation of mesondoderm differentiation and in opposite low Oct4 and high Sox2 levels characterized the beginning of neuroectoderm differentiation [27]. Cell fate selection by decreasing of one of OS factors and increasing another one was also proved by the facts that Oct4 or Sox2 overexpression led to specific differentiation. Oct4 overexpression resulted in differentiation to mesendoderm precursors in the presence of LIF and to ectoderm in the absence of LIF and BMPs [26, 89]. Increase in Sox2 expression triggered mESCs to neuroectoderm and mesoderm [67].

### Positive feedback loops from Nanog to Oct_4_ and Sox_2_ drive the system towards the ESC naïve stage

**Fig 8:**
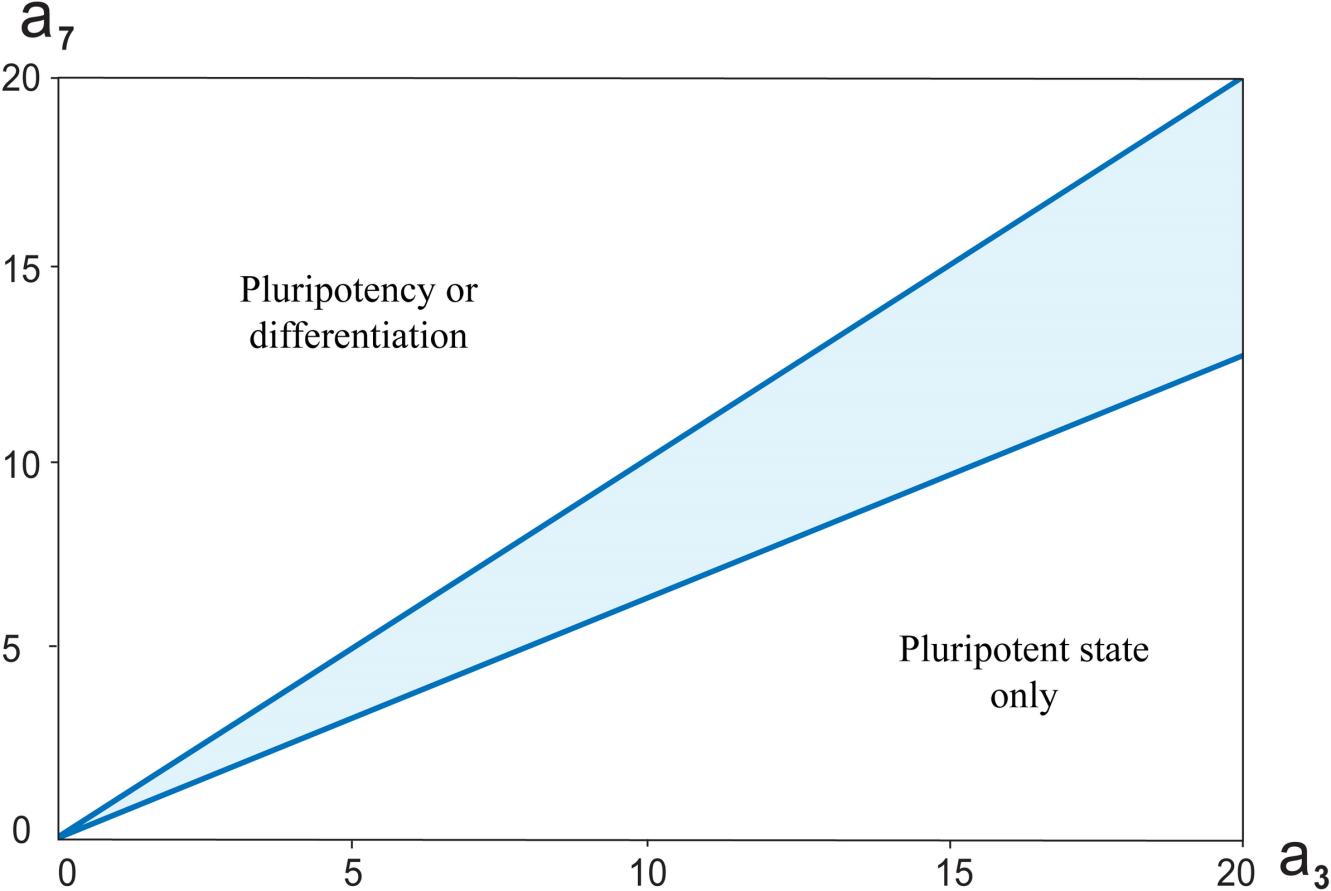
The bistable switch in the core network depending on (*a*_*3*_, *a*_*7*_) parameters and at *h = 6*. Highlighted region is the parameters range, for which the switch-like behavior has existed. Furthermore, the analysis indicated that a straight line *a*_*3*_ = *a*_*7*_ in the plane (*a*_*3*_, *a*_*7*_) divides it into two areas. When *a*_*3*_ < *a*_*7*_, there will be some *A*_*0*_, that upon *A > A*_*0*_ the cell is pluripotent, while at *A < A*_*0*_ the cell will differentiate. When *a*_*3*_ > *a*_*7*_, the cell has pluripotent state at all values *A* ≥ *0*.

Lakatos with coauthors [43] considered in their simulations different variants of OSN transcriptional regulatory circuit, in which Nanog activates or not activates *Oct4* expression; Oct4 represses *Nanog* and Nanog autorepression and combination of two latest regulations. The model in all these modifications demonstrated bistable, switch-like behavior. To verify the significance of the feedback loops from Nanog to *Oct4* and *Sox2* in the revised network we also considered the model extended by an addition of positive feedback loops that led to the update of the system of differential equations (Fig 8; Materials and Methods).

The system also exhibits bistability for the same range of parameter values as not extended variant. Moreover, accounting for the additional positive feedback loops did not result in emergence of the new type of steady states (subsection “Threshold in OS activation defining differentiation-pluripotency transition”). However, it led to significant qualitative change in functional cell state on the plane (*a*_*3*_, *a*_*7*_) (Fig 8). The numerical analysis showed that the straight line *a*_*3*_ = *a*_*7*_ that belong to the wedge-shaped region in the plane (*a*_*3*_, *a*_*7*_) is an approximate boundary between an area *a*_*3*_ < *a*_*7*_, in which both pluripotency and differentiation criteria can be performed upon a certain value of the external A signal and an area *a*_*3*_ > *a*_*7*_, in which the pluripotent state only exists at *A* ≥ *0*.

Therefore, additional activation of *Oct4* and *Sox2* expressions through Nanog positive feedback gives rise to much stronger enhance of the PTG expression simultaneously with increasing of repression effect on DTG expression. This corresponds to naïve pluripotency where differentiation genes are strongly repressed and expression of pluripotency genes is stable and homogeneous [68, 69].

### Concluding remarks

In this work, a new kinetic model of revisited minimal regulatory circuit for mouse pluripotent cell induction, self-renewal and differentiation was proposed. We have conducted a thorough parametric analysis of the developed model. Numerical simulations suggest that the system dynamics is mainly sensitive to variations of two model parameters: *h*_,_ which is a Hill coefficient determining the nonlinear effect of Nanog autorepression and *A* parameter, which is a value of the external signal A for activation of *OS* expression. The model predicts for a mouse pluripotent cell the existence of four dynamical domains with different numbers of stable and unstable steady states, which, as we suppose, can present a developmental progression from ground state ESC to lineage commitment and vice versa. Taken together, the computational study indicates that molecular mechanisms of Nanog regulation and OS activation are the most essential for differentiation/pluripotency transition.

## Materials and methods

### Dynamical model of the revised core pluripotency network

We considered an autonomous system of differential equations representing a dynamical model of the revised core pluripotency network (Fig 1B; S1 File). The model constitutes of three groups of equations with parameters *a*_*i*_, *i = 1*,*2*,*…*,*29*, *A* for the first group, parameters *b*_*i*_, *i = 1*,*2*,*…*,*14*, *h* for the second group and parameters *c*_*i*_, *i = 1*,*2*,*…*,*10*, for the third group. Initial values of the parameters and corresponding references are represented below (Table 1).

The first group of equations reflects concentration changes for both mRNAs and proteins of Oct4 and Sox2, considers changes in Oct4-Sox2 heterodimer level and includes 7 differential equations for *u*_*i*_, *i = 1*,*2*,*…*,*7* This group is independent on other following two groups of equations, but defines dynamical behaviors of variables in these groups. The equation system for the first group is written as:

$$\frac{du_{1}}{dt}=a_{1}\frac{a_{2}+a_{3}A+a_{4}{\left(\frac{u_{3}}{a_{5}}\right)}^{a_{6}}}{1+a_{7}A+a_{8}{\left(\frac{u_{3}}{a_{5}}\right)}^{a_{6}}}-a_{9}u_{1}$$

$$\frac{du_{2}}{dt}=a_{10}\frac{a_{11}+a_{12}A+a_{13}{\left(\frac{u_{3}}{a_{14}}\right)}^{a_{15}}}{1+a_{16}A+a_{17}{\left(\frac{u_{3}}{a_{14}}\right)}^{a_{15}}}-a_{18}u_{2}$$

$$\frac{du_{3}}{dt}=a_{19}u_{4}u_{6}-\left(a_{20}+a_{21}\right)u_{3}$$ (1)

$$\frac{du_{4}}{dt}=a_{21}u_{3}+a_{22}u_{5}-\left(a_{19}u_{6}+a_{23}\right)u_{4}$$

$$\frac{du_{5}}{dt}=a_{24}u_{1}-\left(a_{22}+a_{25}\right)u_{5}$$

$$\frac{du_{6}}{dt}=a_{21}u_{3}+a_{26}u_{7}-\left(a_{19}u_{4}+a_{27}\right)u_{6}$$

$$\frac{du_{7}}{dt}=a_{28}u_{2}-\left(a_{26}+a_{29}\right)u_{7}$$

where *u*_*1*_ –*Oct4* mRNA concentration, *u*_*2*_—*Sox2* mRNA concentration, *u*_*3*_—Oct4-Sox2 heterodimer concentration, *u*_*4*,_ *u*_*6*_ –Oct4 and Sox protein concentrations in the nucleus, correspondingly, *u*_*5*,_ *u*_*7*_ –Oct4 and Sox protein concentrations in the cytoplasm, correspondingly.

The second group representing concentration changes for both *Nanog* mRNA and protein (in the cytoplasm as well as in the nucleus) is described by the system of equations for *v*_*1*,_ *v*_*2*,_ *v*_*3*_ variables:

$$\frac{dv_{1}}{dt}=b_{1}\frac{b_{2}+b_{3}{\left(\frac{u_{3}}{b_{4}}\right)}^{b_{5}}}{1+b_{6}{\left(\frac{u_{3}}{b_{4}}\right)}^{b_{5}}+\left(b_{7}+b_{8}{\left(\frac{u_{3}}{b_{4}}\right)}^{b_{5}}\right){\left(\frac{v_{2}}{b_{9}}\right)}^{h}}-b_{10}v_{1}$$

$$\frac{dv_{2}}{dt}=b_{11}v_{3}-b_{12}v_{2}$$ (2)

$$\frac{dv_{3}}{dt}=b_{13}v_{1}-\left(b_{11}+b_{14}\right)v_{3}$$

where *v*_*1*_- *Nanog* mRNA concentration, *v*_*2*_- Nanog protein concentration in the nucleus, *v*_*3*_- Nanog protein concentration in the cytoplasm.

The last group contains equations describing the dynamic of concentration changes for pluripotency and differentiation factors, *w*_*1*_ and *w*_*2*_, correspondingly:

$$\frac{dw_{1}}{dt}=\frac{c_{1}+\left(c_{2}+c_{3}v_{2}\right)u_{3}}{1+\left(c_{4}+c_{5}v_{2}\right)u_{3}}-c_{6}w_{1}$$ (3)

$$\frac{dw_{2}}{dt}=\frac{c_{7}}{1+\left(c_{8}+c_{9}v_{2}\right)u_{3}}-c_{10}w_{2}$$

The numerical analysis of the steady states based on a method of solution continuation with respect to a parameter allowed for the investigation of functional cell state‘s dependence on the external signal A+ concentration. To define cellular attractors we employed diagrams of stationary solutions that represent dependence of *w*_*1*_ and *w*_*2*_concentrations on parameter *A* and by means of the next criterions:

*w*_*1*_ *> w*_*2*_–criterion for pluripotent state,

*w*_*1*_ *< w*_*2*_–criterion for differentiate state.

### The model extension by accounting of several positive feedback loops in the revised core network

Chickarmane and coauthors [10] showed that positive feedback loops between Oct4-Sox2-Nanog factors in the core transcriptional network give rise to bistable switch-like behavior. To verify the role of the feedback loops in the revised network we considered a model extended by an addition of the positive feedback loops (S1 File). For this the system of differential Eq 1 was written as:

$$\frac{du_{1}}{dt}=a_{1}\frac{a_{2}+a_{3}A+a_{4}{\left(\frac{u_{3}}{a_{5}}\right)}^{a_{6}}+q_{1}{\left(\frac{v_{2}}{p_{1}}\right)}^{h_{1}}}{1+a_{7}A+a_{8}{\left(\frac{u_{3}}{a_{5}}\right)}^{a_{6}}+q_{2}{\left(\frac{v_{2}}{p_{1}}\right)}^{h_{1}}}-a_{9}u_{1}$$

$$\frac{du_{2}}{dt}=a_{10}\frac{a_{11}+a_{12}A+a_{13}{\left(\frac{u_{3}}{a_{14}}\right)}^{a_{15}}+q_{3}{\left(\frac{v_{2}}{p_{2}}\right)}^{h_{2}}}{1+a_{16}A+a_{17}{\left(\frac{u_{3}}{a_{14}}\right)}^{a_{15}}+q_{4}{\left(\frac{v_{2}}{p_{2}}\right)}^{h_{2}}}-a_{18}u_{2}$$

$$\frac{du_{3}}{dt}=a_{19}u_{4}u_{6}-\left(a_{20}+a_{21}\right)u_{3}$$

$$\frac{du_{4}}{dt}=a_{21}u_{3}+a_{22}u_{5}-\left(a_{19}u_{6}+a_{23}\right)u_{4}$$ 4

$$\frac{du_{5}}{dt}=a_{24}u_{1}-\left(a_{22}+a_{25}\right)u_{5}$$

$$\frac{du_{6}}{dt}=a_{21}u_{3}+a_{26}u_{7}-\left(a_{19}u_{4}+a_{27}\right)u_{6}$$

$$\frac{du_{7}}{dt}=a_{28}u_{2}-\left(a_{26}+a_{29}\right)u_{7}$$

where *q*_*1*_, *q*_*2*_, *q*_*3*_, *q*_*4*_, *p*_*1*_, *p*_*2*_, *h*_*1*_, *h*_*2*_ are additional to *a*_*i*_, *b*_*j*_, *c*_*k*_, *A*, *h* parameters introduced for taking into account the positive feedback loops. Equations in the systems (2)-(3) remain unchanged. It should be noted that feedback loops impose interdependencies between the systems (4) and (2) for the extended model.

## Supporting information

S1 File A set of MATLAB files: two model modifications, run file and initial values of the model variables. (ZIP)

## References

[1] Smith A. (2017). Formative pluripotency: the executive phase in a developmental continuum. *Development*, 144(3), 365–373. doi: 10.1242/dev.142679 28143843 10.1242/dev.142679 PMC5430734

[2] Li M., Belmonte J. C. I. (2017). Ground rules of the pluripotency gene regulatory network. *Nature Reviews Genetics*. 10.1038/nrg.2016.156 28045100

[3] Betschinger J. (2016). Charting developmental dissolution of pluripotency. *Journal of Molecular Biology*. 10.1016/j.jmb.2016.12.017 28013029

[4] Makarev E., Fortney K., Litovchenko M., Braunewell K. H., Zhavoronkov A., Atala A. (2015). Quantifying signaling pathway activation to monitor the quality of induced pluripotent stem cells. *Oncotarget*, 6(27), 23204 doi: 10.18632/oncotarget.4673 26327604 10.18632/oncotarget.4673 PMC4695112

[5] van den Hurk M., Kenis G., Bardy C., van den Hove D. L., Gage F. H., Steinbusch H. W., et al (2016). Transcriptional and epigenetic mechanisms of cellular reprogramming to induced pluripotency. *Epigenomics*, 8(8), 1131–1149. doi: 10.2217/epi-2016-0032 27419933 10.2217/epi-2016-0032 PMC5514980

[6] Bilic J., Belmonte J. C. I. (2012). Concise review: Induced pluripotent stem cells versus embryonic stem cells: close enough or yet too far apart?. *Stem Cells*, 30(1), 33–41. doi: 10.1002/stem.700 22213481 10.1002/stem.700

[7] Herberg M., Roeder I. (2015). Computational modelling of embryonic stem-cell fate control. *Development*, 142(13), 2250–2260. doi: 10.1242/dev.116343 26130756 10.1242/dev.116343

[8] Espinosa Angarica V., Del Sol A. (2016). Modeling heterogeneity in the pluripotent state: A promising strategy for improving the efficiency and fidelity of stem cell differentiation. *Bioessays*, 38(8), 758–768. doi: 10.1002/bies.201600103 27321053 10.1002/bies.201600103 PMC5094535

[9] Chen C. Y., Cheng Y. Y., Yen C. Y., Hsieh P. C. (2017). Mechanisms of pluripotency maintenance in mouse embryonic stem cells. *Cellular and Molecular Life Sciences*, 1–13. 10.1007/s00018-016-2438-0 PMC11107721 27999898

[10] Chickarmane V., Troein C., Nuber U. A., Sauro H. M., Peterson C. (2006). Transcriptional dynamics of the embryonic stem cell switch. *PLoS Computational Biology*, 2(9), e123 doi: 10.1371/journal.pcbi.0020123 16978048 10.1371/journal.pcbi.0020123 PMC1570179

[11] Chickarmane V., Peterson C. (2008). A computational model for understanding stem cell, trophectoderm and endoderm lineage determination. *PLoS One*, 3(10), e3478 doi: 10.1371/journal.pone.0003478 18941526 10.1371/journal.pone.0003478 PMC2566811

[12] Chickarmane V., Olariu V., Peterson C. (2012). Probing the role of stochasticity in a model of the embryonic stem cell–heterogeneous gene expression and reprogramming efficiency. *BMC Systems Biology*, 6(1), 98. 22889237 10.1186/1752-0509-6-98 PMC3468383

[13] Boyer L. A., Lee T. I., Cole M. F., Johnstone S. E., Levine S. S., Zucker J. P., et al (2005). Core transcriptional regulatory circuitry in human embryonic stem cells. *Cell*, 122(6), 947–956. doi: 10.1016/j.cell.2005.08.020 16153702 10.1016/j.cell.2005.08.020 PMC3006442

[14] Loh Y. H., Wu Q., Joon-Lin C., Vega V. B., Zhang W., Chen X., et al (2006). The Oct4 and Nanog transcription network regulates pluripotency in mouse embryonic stem cells. *Nature Genetics*, 38(4), 431 doi: 10.1038/ng1760 16518401 10.1038/ng1760

[15] Tomioka M., Nishimoto M., Miyagi S., Katayanagi T., Fukui N., Niwa H., et al (2002). Identification of Sox‐2 regulatory region which is under the control of Oct‐3/4–Sox‐2 complex. *Nucleic Acids Research*, 30(14), 3202–3213. 12136102 10.1093/nar/gkf435 PMC135755

[16] Chew J. L., Loh Y. H., Zhang W., Chen X., Tam W. L., Yeap L. S., et al (2005). Reciprocal transcriptional regulation of Pou5f1 and Sox2 via the Oct4/Sox2 complex in embryonic stem cells. *Molecular and Cellular Biology*, 25(14), 6031–6046. doi: 10.1128/MCB.25.14.6031-6046.2005 15988017 10.1128/MCB.25.14.6031-6046.2005 PMC1168830

[17] Kuroda T., Tada M., Kubota H., Kimura H., Hatano S. Y., Suemori H., et al (2005). Octamer and Sox elements are required for transcriptional cis regulation of Nanog gene expression. *Molecular and Cellular Biology*, 25(6), 2475–2485. doi: 10.1128/MCB.25.6.2475-2485.2005 15743839 10.1128/MCB.25.6.2475-2485.2005 PMC1061601

[18] Okumura-Nakanishi S., Saito M., Niwa H., Ishikawa F. (2005). Oct-3/4 and Sox2 regulate Oct-3/4 gene in embryonic stem cells. *Journal of Biological Chemistry*, 280(7), 5307–5317. doi: 10.1074/jbc.M410015200 15557334 10.1074/jbc.M410015200

[19] Rodda D. J., Chew J. L., Lim L. H., Loh Y. H., Wang B., Ng H. H., et al (2005). Transcriptional regulation of nanog by OCT4 and SOX2. *Journal of Biological Chemistry*, 280(26), 24731–24737. doi: 10.1074/jbc.M502573200 15860457 10.1074/jbc.M502573200

[20] Pan G., Li J., Zhou Y., Zheng H., Pei D. (2006). A negative feedback loop of transcription factors that controls stem cell pluripotency and self-renewal. *The FASEB Journal*, 20(10), 1730–1732. doi: 10.1096/fj.05-5543fje 16790525 10.1096/fj.05-5543fje

[21] Karwacki-Neisius V., Göke J., Osorno R., Halbritter F., Ng J. H., Weiβe A. Y., et al (2013). Reduced Oct4 expression directs a robust pluripotent state with distinct signaling activity and increased enhancer occupancy by Oct4 and Nanog. *Cell Stem Cell*, 12(5), 531–545. doi: 10.1016/j.stem.2013.04.023 23642364 10.1016/j.stem.2013.04.023 PMC3650585

[22] Navarro P., Festuccia N., Colby D., Gagliardi A., Mullin N. P., Zhang W., et al (2012). OCT4/SOX2‐independent Nanog autorepression modulates heterogeneous Nanog gene expression in mouse ES cells. *The EMBO Journal*, 31(24), 4547–4562. doi: 10.1038/emboj.2012.321 23178592 10.1038/emboj.2012.321 PMC3545296

[23] Lewis J. (2003). Autoinhibition with transcriptional delay: a simple mechanism for the zebrafish somitogenesis oscillator. *Current Biology*, 13(16), 1398–1408. 12932323 10.1016/s0960-9822(03)00534-7

[24] Chambers I., Colby D., Robertson M., Nichols J., Lee S., Tweedie S., et al (2003). Functional expression cloning of Nanog, a pluripotency sustaining factor in embryonic stem cells. *Cell*, 113(5), 643–655. 12787505 10.1016/s0092-8674(03)00392-1

[25] Mitsui K., Tokuzawa Y., Itoh H., Segawa K., Murakami M., Takahashi K., et al (2003). The homeoprotein Nanog is required for maintenance of pluripotency in mouse epiblast and ES cells. *Cell*, 113(5), 631–642. 12787504 10.1016/s0092-8674(03)00393-3

[26] Niwa H., Miyazaki J. I., Smith A. G. (2000). Quantitative expression of Oct-3/4 defines differentiation, dedifferentiation or self-renewal of ES cells. *Nature Genetics*, 24(4), 372 doi: 10.1038/74199 10742100 10.1038/74199

[27] Thomson M., Liu S. J., Zou L. N., Smith Z., Meissner A., Ramanathan S. (2011). Pluripotency factors in embryonic stem cells regulate differentiation into germ layers. *Cell*, 145(6), 875–889. doi: 10.1016/j.cell.2011.05.017 21663792 10.1016/j.cell.2011.05.017 PMC5603300

[28] Wang J., Zhang Y., Hou J., Qian X., Zhang H., Zhang Z., et al (2016). Ube2s regulates Sox2 stability and mouse ES cell maintenance. *Cell Death and Differentiation*, 23(3), 393–404. doi: 10.1038/cdd.2015.106 26292759 10.1038/cdd.2015.106 PMC5072435

[29] Takahashi K., Yamanaka S. (2006). Induction of pluripotent stem cells from mouse embryonic and adult fibroblast cultures by defined factors. *Cell*, 126(4), 663–676. doi: 10.1016/j.cell.2006.07.024 16904174 10.1016/j.cell.2006.07.024

[30] Silva J., Chambers I., Pollard S., Smith A. (2006). Nanog promotes transfer of pluripotency after cell fusion. *Nature*, 441(7096), 997 doi: 10.1038/nature04914 16791199 10.1038/nature04914

[31] Hanna J., Saha K., Pando B., Van Zon J., Lengner C. J., Creyghton M. P., et al (2009). Direct cell reprogramming is a stochastic process amenable to acceleration. *Nature*, 462(7273), 595 doi: 10.1038/nature08592 19898493 10.1038/nature08592 PMC2789972

[32] Brambrink T., Foreman R., Welstead G. G., Lengner C. J., Wernig M., Suh H., et al (2008). Sequential expression of pluripotency markers during direct reprogramming of mouse somatic cells. *Cell Stem Cell*, 2(2), 151–159. doi: 10.1016/j.stem.2008.01.004 18371436 10.1016/j.stem.2008.01.004 PMC2276627

[33] Silva J., Nichols J., Theunissen T. W., Guo G., van Oosten A. L., Barrandon O., et al (2009). Nanog is the gateway to the pluripotent ground state. *Cell*, 138(4), 722–737. doi: 10.1016/j.cell.2009.07.039 19703398 10.1016/j.cell.2009.07.039 PMC3437554

[34] Fidalgo M., Faiola F., Pereira C. F., Ding J., Saunders A., Gingold J., et al (2012). Zfp281 mediates Nanog autorepression through recruitment of the NuRD complex and inhibits somatic cell reprogramming. *Proceedings of the National Academy of Sciences*, 109(40), 16202–16207. 10.1073/pnas.1208533109 PMC3479613 22988117

[35] Chambers I., Silva J., Colby D., Nichols J., Nijmeijer B., Robertson M., et al (2007). Nanog safeguards pluripotency and mediates germline development. *Nature*, 450(7173), 1230 doi: 10.1038/nature06403 18097409 10.1038/nature06403

[36] Kalmar T., Lim C., Hayward P., Muñoz-Descalzo S., Nichols J., Garcia-Ojalvo J., et al (2009). Regulated fluctuations in Nanog expression mediate cell fate decisions in embryonic stem cells. *PLoS Biology*, 7(7), e1000149 doi: 10.1371/journal.pbio.1000149 19582141 10.1371/journal.pbio.1000149 PMC2700273

[37] Singer Z. S., Yong J., Tischler J., Hackett J. A., Altinok A., Surani M. A., et al (2014). Dynamic heterogeneity and DNA methylation in embryonic stem cells. *Molecular Cell*, 55(2), 319–331. doi: 10.1016/j.molcel.2014.06.029 25038413 10.1016/j.molcel.2014.06.029 PMC4104113

[38] Filipczyk A., Marr C., Hastreiter S., Feigelman J., Schwarzfischer M., Hoppe P. S., et al (2015). Network plasticity of pluripotency transcription factors in embryonic stem cells. *Nature Cell Biology*, 17(10), 1235 doi: 10.1038/ncb3237 26389663 10.1038/ncb3237

[39] Hatano S. Y., Tada M., Kimura H., Yamaguchi S., Kono T., Nakano T., et al (2005). Pluripotential competence of cells associated with Nanog activity. *Mechanisms of Development*, 122(1), 67–79. doi: 10.1016/j.mod.2004.08.008 15582778 10.1016/j.mod.2004.08.008

[40] Glauche I., Herberg M., Roeder I. (2010). Nanog variability and pluripotency regulation of embryonic stem cells-insights from a mathematical model analysis. *PloS One*, 5(6), e11238 doi: 10.1371/journal.pone.0011238 20574542 10.1371/journal.pone.0011238 PMC2888652

[41] MacArthur B. D., Please C. P., Oreffo R. O. (2008). Stochasticity and the molecular mechanisms of induced pluripotency. *PloS One*, 3(8), e3086 doi: 10.1371/journal.pone.0003086 18769478 10.1371/journal.pone.0003086 PMC2517845

[42] Wu Q., Jiang F., Tian T. (2015). Sensitivity and Robustness Analysis for Stochastic Model of Nanog Gene Regulatory Network. *International Journal of Bifurcation and Chaos*, 25(07), 1540009.

[43] Lakatos D., Travis E. D., Pierson K. E., Vivian J. L., Czirok A. (2014). Autocrine FGF feedback can establish distinct states of Nanog expression in pluripotent stem cells: a computational analysis. *BMC Systems Biology*, 8(1), 112. 25267505 10.1186/s12918-014-0112-4 PMC4189679

[44] Abranches E., Guedes A. M., Moravec M., Maamar H., Svoboda P., Raj A., et al (2014). Stochastic NANOG fluctuations allow mouse embryonic stem cells to explore pluripotency. *Development*, 141(14), 2770–2779. doi: 10.1242/dev.108910 25005472 10.1242/dev.108910 PMC6517831

[45] Fadeev S. I., Kogai V. V. (2004). Using parameter continuation based on the multiple shooting method for numerical research of nonlinear boundary value problems. *International Journal of Pure and Applied Mathematics*, 14, 467–498.

[46] Saxe J. P., Tomilin A., Schöler H. R., Plath K., Huang J. (2009). Post-translational regulation of Oct4 transcriptional activity. *PloS One*, 4(2), e4467 doi: 10.1371/journal.pone.0004467 19221599 10.1371/journal.pone.0004467 PMC2637973

[47] Abranches E., Bekman E., Henrique D. (2013). Generation and characterization of a novel mouse embryonic stem cell line with a dynamic reporter of Nanog expression. *PLoS One*, 8(3), e59928 doi: 10.1371/journal.pone.0059928 23527287 10.1371/journal.pone.0059928 PMC3602340

[48] Likhoshvai V., Ratushny A. (2007). Generalized Hill function method for modeling molecular processes. *Journal of Bioinformatics and Computational Biology*, 5(02b), 521–531. 17636859 10.1142/s0219720007002837

[49] Fadeev, S. I., Korolev, V. K., Gainova, I. A., Medvedev, A. E. (2006, July). The package Step+ for numerical study of autonomous systems arising when modeling dynamics of genetic-molecular systems. In Proc. of the 6th Intern. Conf. on Bioinformatics of Genome Regulation and Structure (Vol. 118, p. 120).

[50] Kholodniok M, Klich A, Kubichek M, Marek M. Methods for Analysis of Nonlinear Dynamic Models [Russian translation], Mir, Moscow (1991).

[51] Buganim Y., Faddah D. A., Cheng A. W., Itskovich E., Markoulaki S., Ganz K., et al (2012). Single-cell expression analyses during cellular reprogramming reveal an early stochastic and a late hierarchic phase. *Cell*, 150(6), 1209–1222. doi: 10.1016/j.cell.2012.08.023 22980981 10.1016/j.cell.2012.08.023 PMC3457656

[52] Radzisheuskaya A., Chia G. L. B., Dos Santos R. L., Theunissen T. W., Castro L. F. C., Nichols J., et al (2013). A defined Oct4 level governs cell state transitions of pluripotency entry and differentiation into all embryonic lineages. *Nature Cell Biology*, 15(6), 579 doi: 10.1038/ncb2742 23629142 10.1038/ncb2742 PMC3671976

[53] Mullin N.P., Yates A., Rowe A.J., Nijmeijer B., Colby D., Barlow P.N., et al (2008). The pluripotency rheostat Nanog functions as a dimer. *Biochemical Journal*, 411(2), 227–31. doi: 10.1042/BJ20080134 18290762 10.1042/BJ20080134

[54] Wang J., Levasseur D. N., Orkin S. H. (2008). Requirement of Nanog dimerization for stem cell self-renewal and pluripotency. *Proceedings of the National Academy of Sciences*, 105(17), 6326–6331. 10.1073/pnas.0802288105 PMC2359802 18436640

[55] Suzuki A., Raya A., Kawakami Y., Morita M., Matsui T., Nakashima K., et al (2006). Nanog binds to Smad1 and blocks bone morphogenetic protein-induced differentiation of embryonic stem cells. *Proceedings of the National Academy of Sciences*, 103(27), 10294–10299. 10.1073/pnas.0506945103 PMC1502451 16801560

[56] MacArthur B. D., Sevilla A., Lenz M., Müller F. J., Schuldt B. M., Schuppert A. A., et al (2012). Nanog-dependent feedback loops regulate murine embryonic stem cell heterogeneity. *Nature Cell Biology*, 14(11), 1139 doi: 10.1038/ncb2603 23103910 10.1038/ncb2603 PMC3507454

[57] Gingold J. A., Fidalgo M., Guallar D., Lau Z., Sun Z., Zhou H., et al (2014). A genome-wide RNAi screen identifies opposing functions of Snai1 and Snai2 on the Nanog dependency in reprogramming. *Molecular Cell*, 56(1), 140–152. doi: 10.1016/j.molcel.2014.08.014 25240402 10.1016/j.molcel.2014.08.014 PMC4184964

[58] Boroviak T., Loos R., Lombard P., Okahara J., Behr R., Sasaki E., et al (2015). Lineage-specific profiling delineates the emergence and progression of naive pluripotency in mammalian embryogenesis. *Developmental Cell*, 35(3), 366–382. doi: 10.1016/j.devcel.2015.10.011 26555056 10.1016/j.devcel.2015.10.011 PMC4643313

[59] Kalkan T., Olova N., Roode M., Mulas C., Lee H. J., Nett I., et al (2017). Tracking the embryonic stem cell transition from ground state pluripotency. *Development*, dev-142711. 10.1242/dev.142711 PMC5399622 28174249

[60] Osorno R., Chambers I. (2011). Transcription factor heterogeneity and epiblast pluripotency. *Philosophical Transactions of the Royal Society of London B*: *Biological Sciences*, 366(1575), 2230–2237. doi: 10.1098/rstb.2011.0043 21727128 10.1098/rstb.2011.0043 PMC3130424

[61] Martello G., Smith A. (2014). The nature of embryonic stem cells. *Annual Review of Cell and Developmental Biology*, 30. 10.1146/annurev-cellbio-100913-013116 25288119

[62] D'Aniello C., Habibi E., Cermola F., Paris D., Russo F., Fiorenzano A., et al (2017). Vitamin C and l-proline antagonistic effects capture alternative states in the pluripotency continuum. *Stem Cell Reports*, 8(1), 1–10. doi: 10.1016/j.stemcr.2016.11.011 28017658 10.1016/j.stemcr.2016.11.011 PMC5233408

[63] Theunissen T. W., Silva J. C. (2012). Somatic Cell Reprogramming: Role of Homeodomain Protein Nanog In *Stem Cells and Cancer Stem Cells*, *Volume* 6(pp. 377–384). Springer Netherlands.

[64] Komatsu K., Fujimori T. (2015). Multiple phases in regulation of Nanog expression during pre‐implantation development. *Development*, *Growth & Differentiation*, 57(9), 648–656. 10.1111/dgd.12244 26660234

[65] Nichols J., Smith A. (2009). Naive and primed pluripotent states. *Cell Stem Cell*, 4(6), 487–492. doi: 10.1016/j.stem.2009.05.015 19497275 10.1016/j.stem.2009.05.015

[66] Stadtfeld M., Maherali N., Breault D. T., Hochedlinger K. (2008). Defining molecular cornerstones during fibroblast to iPS cell reprogramming in mouse. *Cell stem cell*, 2(3), 230–240. doi: 10.1016/j.stem.2008.02.001 18371448 10.1016/j.stem.2008.02.001 PMC3538379

[67] Kopp J. L., Ormsbee B. D., Desler M., Rizzino A. (2008). Small increases in the level of Sox2 trigger the differentiation of mouse embryonic stem cells. *Stem Cells*, 26(4), 903–911. doi: 10.1634/stemcells.2007-0951 18238855 10.1634/stemcells.2007-0951

[68] Wray, J., Kalkan, T., Smith, A. G. (2010). The ground state of pluripotency. 10.1042/BST0381027 20658998

[69] Marks H., Kalkan T., Menafra R., Denissov S., Jones K., Hofemeister H., et al (2012). The transcriptional and epigenomic foundations of ground state pluripotency. *Cell*, 149(3), 590–604. doi: 10.1016/j.cell.2012.03.026 22541430 10.1016/j.cell.2012.03.026 PMC3398752

[70] Miyamoto T., Furusawa C., Kaneko K. (2015). Pluripotency, differentiation, and reprogramming: a gene expression dynamics model with epigenetic feedback regulation. *PLoS Computational Biology*, 11(8), e1004476 doi: 10.1371/journal.pcbi.1004476 26308610 10.1371/journal.pcbi.1004476 PMC4550282

[71] Silva J., Barrandon O., Nichols J., Kawaguchi J., Theunissen T. W., Smith A. (2008). Promotion of reprogramming to ground state pluripotency by signal inhibition. *PLoS Biology*, 6(10), e253 doi: 10.1371/journal.pbio.0060253 18942890 10.1371/journal.pbio.0060253 PMC2570424

[72] Furusawa T., Ikeda M., Inoue F., Ohkoshi K., Hamano T., Tokunaga T. (2006). Gene expression profiling of mouse embryonic stem cell subpopulations. *Biology of reproduction*, 75(4), 555–561. doi: 10.1095/biolreprod.105.049502 16687650 10.1095/biolreprod.105.049502

[73] Hayashi K., de Sousa Lopes S. M. C., Tang F., Surani M. A. (2008). Dynamic equilibrium and heterogeneity of mouse pluripotent stem cells with distinct functional and epigenetic states. *Cell Stem Cell*, 3(4), 391–401. doi: 10.1016/j.stem.2008.07.027 18940731 10.1016/j.stem.2008.07.027 PMC3847852

[74] Toyooka Y., Shimosato D., Murakami K., Takahashi K., Niwa H. (2008). Identification and characterization of subpopulations in undifferentiated ES cell culture. *Development*, 135(5), 909–918. doi: 10.1242/dev.017400 18263842 10.1242/dev.017400

[75] Galvin‐Burgess K. E., Travis E. D., Pierson K. E., Vivian J. L. (2013). TGF‐β‐Superfamily Signaling Regulates Embryonic Stem Cell Heterogeneity: Self‐Renewal as a Dynamic and Regulated Equilibrium. *Stem Cells*, 31(1), 48–58. doi: 10.1002/stem.1252 23081664 10.1002/stem.1252 PMC3528825

[76] Herberg M., Kalkan T., Glauche I., Smith A., Roeder I. (2014). A model-based analysis of culture-dependent phenotypes of mESCs. *PloS One*, 9(3), e92496 doi: 10.1371/journal.pone.0092496 24643025 10.1371/journal.pone.0092496 PMC3958526

[77] Pigolotti S., Krishna S., Jensen M. H. (2007). Oscillation patterns in negative feedback loops. *Proceedings of the National Academy of Sciences*, 104(16), 6533–6537. 10.1073/pnas.0610759104 PMC1871820 17412833

[78] Chen X., Xu H., Yuan P., Fang F., Huss M., Vega V. B., et al (2008). Integration of external signaling pathways with the core transcriptional network in embryonic stem cells. *Cell*, 133(6), 1106–1117. doi: 10.1016/j.cell.2008.04.043 18555785 10.1016/j.cell.2008.04.043

[79] Chen J., Liu H., Liu J., Qi J., Wei B., Yang J., et al (2013). H3K9 methylation is a barrier during somatic cell reprogramming into iPSCs. *Nature Genetics*, 45(1), 34–42. doi: 10.1038/ng.2491 23202127 10.1038/ng.2491

[80] Ji P., Manupipatpong S., Xie N., Li Y. (2016). Induced pluripotent stem cells: generation strategy and epigenetic mystery behind reprogramming. *Stem Cells International*, 2016. 10.1155/2016/8415010 PMC4736417 26880993

[81] Fernandes M. G., Dries R., Roost M. S., Semrau S., de Melo Bernardo A., Davis R. P., et al (2016). BMP-SMAD signaling regulates lineage priming, but is dispensable for self-renewal in mouse embryonic stem cells. *Stem Cell Reports*, 6(1), 85–94. doi: 10.1016/j.stemcr.2015.11.012 26711875 10.1016/j.stemcr.2015.11.012 PMC4720007

[82] Shimojo H., Ohtsuka T., Kageyama R. (2008). Oscillations in Notch signaling regulate maintenance of neural progenitors. *Neuron*, 58(1), 52–64. doi: 10.1016/j.neuron.2008.02.014 18400163 10.1016/j.neuron.2008.02.014

[83] Imayoshi I., Isomura A., Harima Y., Kawaguchi K., Kori H., Miyachi H., et al (2013). Oscillatory control of factors determining multipotency and fate in mouse neural progenitors. *Science*, 342(6163), 1203–1208. doi: 10.1126/science.1242366 24179156 10.1126/science.1242366

[84] Furusawa C., Kaneko K. (2012). A dynamical-systems view of stem cell biology. *Science*, 338(6104), 215–217. doi: 10.1126/science.1224311 23066073 10.1126/science.1224311

[85] Shu J., Wu C., Wu Y., Li Z., Shao S., Zhao W., et al (2013). Induction of pluripotency in mouse somatic cells with lineage specifiers. *Cell*, 153(5), 963–975. doi: 10.1016/j.cell.2013.05.001 23706735 10.1016/j.cell.2013.05.001 PMC4640445

[86] Montserrat N., Nivet E., Sancho-Martinez I., Hishida T., Kumar S., Miquel L., et al (2013). Reprogramming of human fibroblasts to pluripotency with lineage specifiers. *Cell Stem Cell*, 13(3), 341–350. doi: 10.1016/j.stem.2013.06.019 23871606 10.1016/j.stem.2013.06.019

[87] Hahn S., Hermeking H. (2014). ZNF281/ZBP-99: a new player in epithelial–mesenchymal transition, stemness, and cancer. *Journal of Molecular Medicine*, 92(6), 571–581. doi: 10.1007/s00109-014-1160-3 24838609 10.1007/s00109-014-1160-3

[88] Herberg M., Glauche I., Zerjatke T., Winzi M., Buchholz F., Roeder I. (2016). Dissecting mechanisms of mouse embryonic stem cells heterogeneity through a model-based analysis of transcription factor dynamics. *Journal of The Royal Society Interface*, 13(117), 20160167. 10.1098/rsif.2016.0167 PMC4874438 27097654

[89] Shimozaki K., Nakashima K., Niwa H., Taga T. (2003). Involvement of Oct3/4 in the enhancement of neuronal differentiation of ES cells in neurogenesis-inducing cultures. *Development*, 130(11), 2505–2512. 12702663 10.1242/dev.00476

